# Automated radiosynthesis and preclinical imaging of a novel [^18^F]fluorolidocaine analogue *via* sequential C–H radiolabelling†

**DOI:** 10.1039/d4md00293h

**Published:** 2024-07-31

**Authors:** Madison Frazier, Jay S. Wright, David M. Raffel, Jenelle Stauff, Wade P. Winton, Peter J. H. Scott, Allen F. Brooks

**Affiliations:** a Department of Radiology, University of Michigan Ann Arbor Michigan 48109 USA jawr@med.umich.edu pjhscott@med.umich.edu afb@med.umich.edu

## Abstract

The most prominent myocardial voltage-gated sodium channel, Na_V_1.5, is a major drug target for treating cardiovascular disease. However, treatment determination and therapeutic development are complicated partly by an inadequate understanding of how the density of SCN5A, the gene that encodes Na_V_1.5, relates to treatment response and disease prognosis. To address these challenges, imaging agents derived from Na_V_1.5 blocking therapeutics have been employed in positron emission tomography (PET) imaging to infer how SCN5A expression relates to human disease *in vivo*. Herein, we describe the preparation of a novel fluorine-18 labelled analogue of lidocaine, a known Na_V_1.5 inhibitor, and compare this agent to a previously described analogue. Evidence from rodent and non-human primate PET imaging experiments suggests that the imaging utility of these agents may be limited by rapid metabolism and clearance.

## Abstract 2

A new fluorine-18-labelled lidocaine analogue has been successfully prepared using sequential Ir/Cu C–H radiofluorination. Preclinical PET studies indicate that these imaging agents undergo rapid metabolism and clearance.

## Graphical abstract

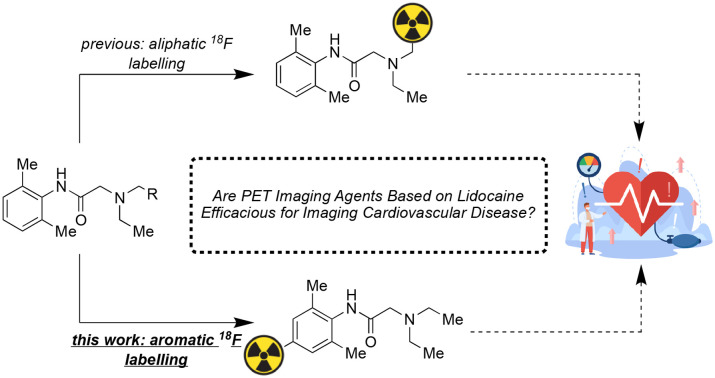

## Introduction

**Fig. 1:**
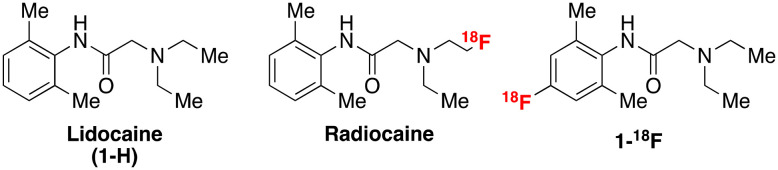
Chemical structures of lidocaine, radiocaine (Hooker), and **1-**^**18**^**F** (this study).

Voltage-gated sodium channels, specifically Na_V_1.5 located in the cardiac myocytes, are key drug targets in treating various diseases of the cardiovascular system, including heart failure, angina, and arrhythmia.^1^ The most common and lethal forms of arrhythmia result from a mutation in the SCN5A gene, which encodes the Na_V_1.5 channel subunits. This mutation often results in ineffective fast inactivation and induces a characteristically abnormal heart rhythm with potentially life-threatening outcomes. This mutation may also affect the efficacy of some antiarrhythmic agents due to changes in Na channel concentration and function. Therefore, using labelled analogues of these antiarrhythmic agents, such as amino amide anaesthetic derivatives, in conjunction with positron emission tomography (PET) imaging may provide a tool for assessing channel occupancy. These analyses can facilitate the efficacy evaluation of related therapeutics in diseases with altered Na_V_1.5 density.^2,3^ For example, Hooker and co-workers reported promising preclinical studies with a novel labelled radioligand, radiocaine, for myocardial Na channel imaging.^4^ Radiocaine is structurally based on lidocaine (**1-H**), a class Ib antiarrhythmic agent and Na_V_1.5 inhibitor (Fig. 1).

Owing to the reported efficacy of this tracer, and since we have recently reported facile radiolabelling of the lidocaine scaffold *via* new chemistry developed in our group, we opted to conduct further preclinical investigations to establish the metabolic properties of fluorine-18 labelled lidocaine derivatives. Notably, lidocaine undergoes a well-understood metabolic pathway beginning with oxidative *N*-dealkylation of the tertiary amine by cytochrome-p450 enzymes (CYP). Radiocaine, containing a sp^3^ C–^18^F label at the terminus of an ethyl chain (Fig. 1), may be susceptible to an analogous dealkylation, thereby eliminating the key fluorine-18 label. Therefore, it is currently unclear whether the myocardial uptake of radiocaine originates solely from the parent imaging agent or from metabolites that exhibit non-specific binding, which could confound Na channel evaluation *via* PET. We sought to test this hypothesis by investigating **1-**^**18**^**F**, an analogue of radiocaine labelled on the aromatic ring and previously synthesised by our laboratories by sequential C–H radiofluorination.^5^

Herein, we describe the development of a fully automated radiosynthesis of lidocaine derivative **1-**^**18**^**F**, labelled as an aromatic radiofluoride *via* sequential iridium-catalysed C–H borylation and copper-mediated radiofluorination. This radiosynthetic protocol enables rapid access to sufficient **1-**^**18**^**F** for preclinical evaluation. It was hypothesised that CYP dealkylation of **1-**^**18**^**F**, in analogy to the metabolic dealkylation of **1-H**, would afford an alternative distribution of radiolabelled metabolites to radiocaine, which could be visualised *in vivo via* PET. Therefore, we evaluated the preclinical efficacy of this agent in rodents and non-human primates (NHP). These imaging studies elucidate the utility of fluorine-18 labelled lidocaine scaffolds as potential radiopharmaceuticals for evaluating myocardial Na channel density and monitoring cardiovascular disease progression and treatment.

## Discussion

**Scheme 1:**
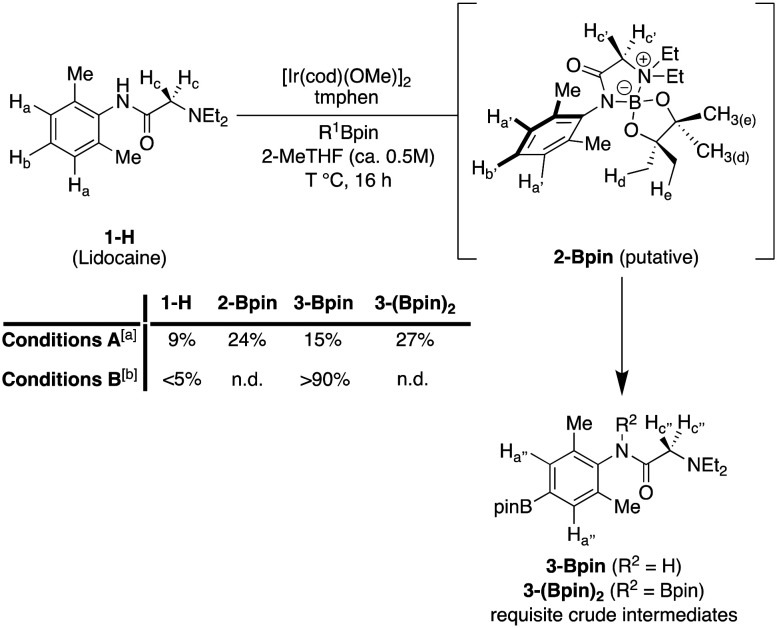
Ir-catalysed C–H borylation of lidocaine **1-H** preceded by rapid N–H borylation. [a] Conditions A: 1 mol% [Ir(cod)(OMe)]_2_, 2 mol% tmphen, 1.8 eq. pinacolborane (HBpin, R^1^ = H), *T* = 80 °C with 0.14 mmol **1-H** in 2-MeTHF (*ca.* 0.5 M). [b] Conditions B: 2 mol% = [Ir(cod)(OMe)]_2_, 4 mol% tmphen, 2.2 eq. bis(pinacolato)diboron (B_2_pin_2_, R^1^ = Bpin), *T* = 100 °C with 0.50 mmol **1-H**.

We identified sequential Ir/Cu C–H radiofluorination as a screening tool to rapidly synthesise **1-**^**18**^**F** for preclinical imaging studies, which our laboratories previously prepared under manual conditions on a reduced scale.^5^ This protocol delivers fluorine-18 labelled arenes from the corresponding aromatic C–H precursors under sterically controlled regioselectivity whilst circumventing isolation of the aryl boronate intermediate. However, unlike other precursors investigated in this study, the manual radiolabelling of **1-H** was relatively inefficient. Indeed, initial attempts to scale this manual reaction up in an automated setting with increased amounts of radioactivity afforded trace quantities of **1-**^**18**^**F**, precluding imaging studies. Accordingly, several structural features of **1-H** were identified as potential impediments to efficiently generating **1-**^**18**^**F**, necessitating a modified radiolabelling procedure. For example, the low yield of **1-**^**18**^**F** was attributed to insufficient crude boronate precursor in the automated copper-mediated reaction owing to low Ir C–H borylation (CHB) conversion of **1-H** using conditions A developed in our original report (1 mol% (1,5-cyclooctadiene)(methoxy)iridium(i) dimer ([Ir(cod)(OMe)]_2_), 2 mol% 3,4,7,8-tetramethyl-1,10-phenanthroline (tmphen), 1.8 eq. pinacolborane (HBpin), at 80 °C with 0.14 mmol **1-H** in 2-MeTHF (*ca.* 0.5 M) for 16 h, see Scheme 1, conditions A). Specifically, many N–H bonds, including those featuring in 2° amides, typically undergo Ir borylation with rates that exceed aromatic C–H borylation, sequestering the HBpin reagent.^6,7^ Under CHB conditions A, aliquots of the reaction were analysed by ^1^H NMR with 1,3,5-trimethoxybenzene as an internal standard. Substantial conversion of **1-H** to multiple products was evident, including a new species containing aromatic proton signals (Scheme 1, H_a′_, H_b′_) shifted upfield relative to **1-H** (H_a_, H_b_) in 24% conversion. Conversely, boryl substituents typically exert a diagnostic deshielding effect on aromatic *ortho-*substituents like protons, causing a downfield NMR signal shift. Indeed, two downfield signals consistent with C–H borylation, tentatively assigned as **3-Bpin** and **3-(Bpin)**_**2**_, were also observed in a total conversion of 42%. In contrast, this new upfield signal did not correspond to any CHB products and was attributed instead to the formation of *N*-boryl adduct **2-Bpin**. This adduct was not purified and unambiguously characterised owing to its high hydrolytic lability and reversion to **1-H**, although several observations provide evidence for the tentative structural assignment of **2-Bpin**. First, N–H borylation by HBpin is accelerated by tertiary amines (*e.g.*, Et_3_N), so **1-H** may catalyse this process. Second, precipitation of a moderately insoluble off-white solid was observed over the course of the reaction. Therefore, N–H borylation of **1-H** may induce intramolecular cyclisation to a B–N ylide with reduced solubility. Third, the α-carbonyl protons (H_c_) in **1-H** are shifted significantly downfield in **2-Bpin** (H_c′_), consistent with a deshielding effect induced by positive charge build-up at the tertiary amine nitrogen. Fourth, the typically ^1^H NMR equivalent geminal dimethyl groups at the Bpin substituent are desymmetrised into a pair of 6H singlets (H_d_ and H_e_), likely arising from the conformational rigidity of **2-Bpin** (see ESI† for further evidence and discussion of the borylation of **1-H**).

Therefore, conditions that would more efficiently activate the C–H_b_ bond of **1-H** were sought. It was hypothesised that elevated temperature could improve the solubility of **2-Bpin** and allow for C–H borylation in improved conversions. To our delight, doubling the loading of the precatalyst components, increasing the temperature from 80 °C to 100 °C, and switching HBpin with the more reactive B_2_pin_2_ (2.20 equivalents), CHB Conditions B, afforded **3-Bpin** as the major product in >90% conversion as determined *via*^1^H NMR spectroscopy. Notably, precipitation was not observed over the course of this reaction. Furthermore, the aromatic protons in **3-Bpin** (H_a′′_) are, as expected, shifted downfield relative to the aromatic protons of **1-H** (H_a_) in the crude ^1^H NMR spectrum, originating from the *ortho*-deshielding effect of the aromatic boryl group. Furthermore, by analysing the crude reaction mixture under inert conditions, we could also clearly observe diborylated **3-(Bpin)**_**2**_ as an additional product of this reaction. Treatment of this crude reaction mixture with EtOH decomposed **3-(Bpin)**_**2**_, leaving **3-Bpin** as the sole product, corresponding to N–B alcoholysis.

**Scheme 2:**
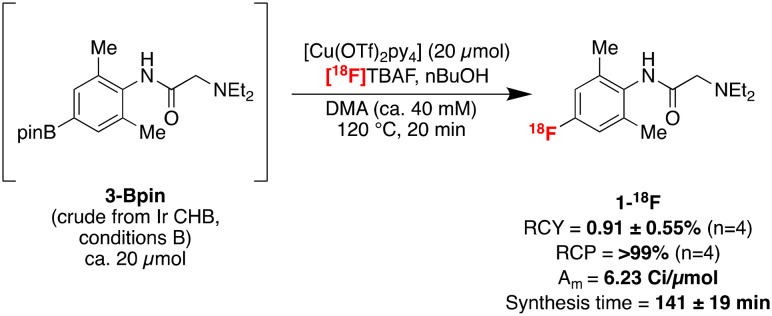
Automated radiosynthesis of **1-**^**18**^**F**.

The automated labelling of **1-H** was next investigated with the optimised CHB conditions. Pleasingly, **1-**^**18**^**F** could be prepared for preclinical imaging studies using a modified labelling protocol (Scheme 2). This first involved independently preparing crude **3-Bpin** from **1-H** under manual Ir-catalysed CHB conditions B. Next, [^18^F]TBAF was prepared from cyclotron-produced ^18^F^−^*via* an azeotropic dry-down in a commercial radiosynthesis module (GE TRACERlab FX_FN_). [Cu(OTf)_2_py_4_] dissolved in DMA, followed by an aliquot of the crude CHB mixture containing boronate **3-Bpin** and *n*BuOH dissolved in DMA, were added successively to the reactor.^8^ The reaction mixture was heated at 120 °C for 20 min, followed by semi-preparative purification with a reverse phase column (Kinetex F5 5 μm, 100 Å, 250 × 10 mm) using 10 mM NH_4_HCO_3_ buffer in 30% MeCN/H_2_O at pH 10. Reformulation with a C18 Sep-Pak cartridge afforded 17 ± 10 mCi (629 ± 370 mBq) **1-**^**18**^**F** in 0.91 ± 0.55% (*n* = 4) isolated non-decay-corrected (ndc) radiochemical yield (RCY) and >99% radiochemical purity (RCP), determined by radio-high-performance liquid chromatography. **1-**^**18**^**F** was also obtained in 3.76 Ci μmol^−1^ ndc molar activity (*A*_m_) with a 141 ± 19 min synthesis time (*n* = 4, see ESI† for full details).

**Fig. 2:**
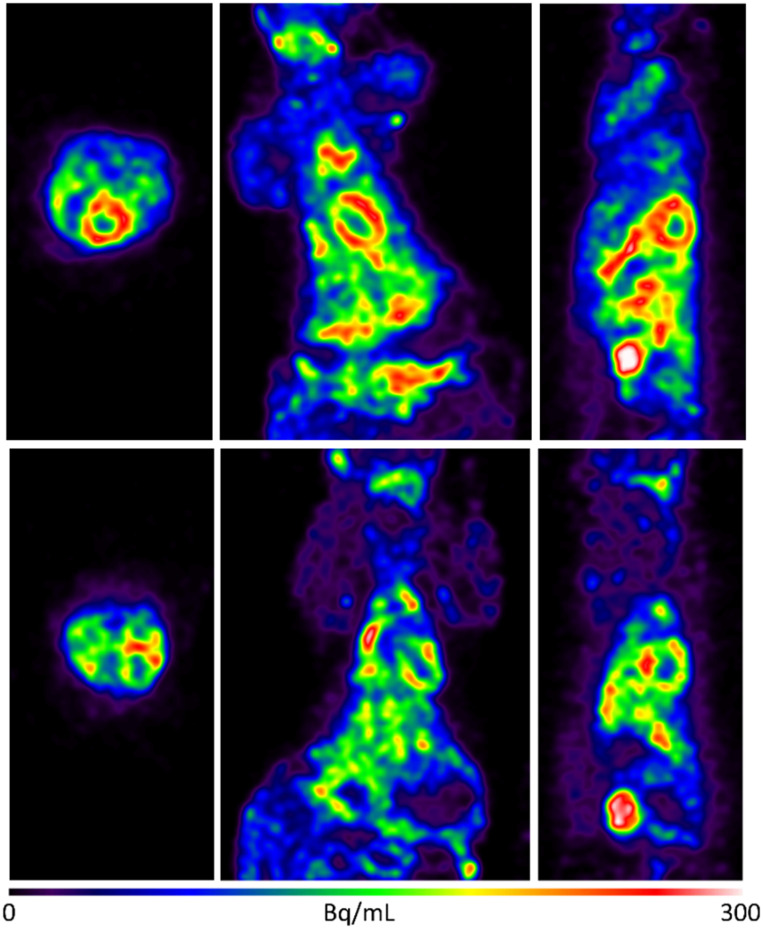
Selected rodent PET imaging data obtained using **1-**^**18**^**F**. Upper: Rodent total summed image frames with rainbow colour table maximum intensity projection baseline control study. Lower: Rodent total summed image frames with rainbow scale table maximum intensity projection – blocking study with **1-H**. Very rapid clearance of **1-**^**18**^**F** was observed from the heart (*ca.* 2 min post-injection) and a significant uptake in the kidneys and bladder, which is consistent with the generation of metabolites such as **[**^**18**^**F]FMEGX** and **[**^**18**^**F]FGX**. Two of the rats received an injection of 2 mg mL^−1^ of lidocaine immediately before imaging for blocking studies. See ESI† for further details.

**Fig. 3:**
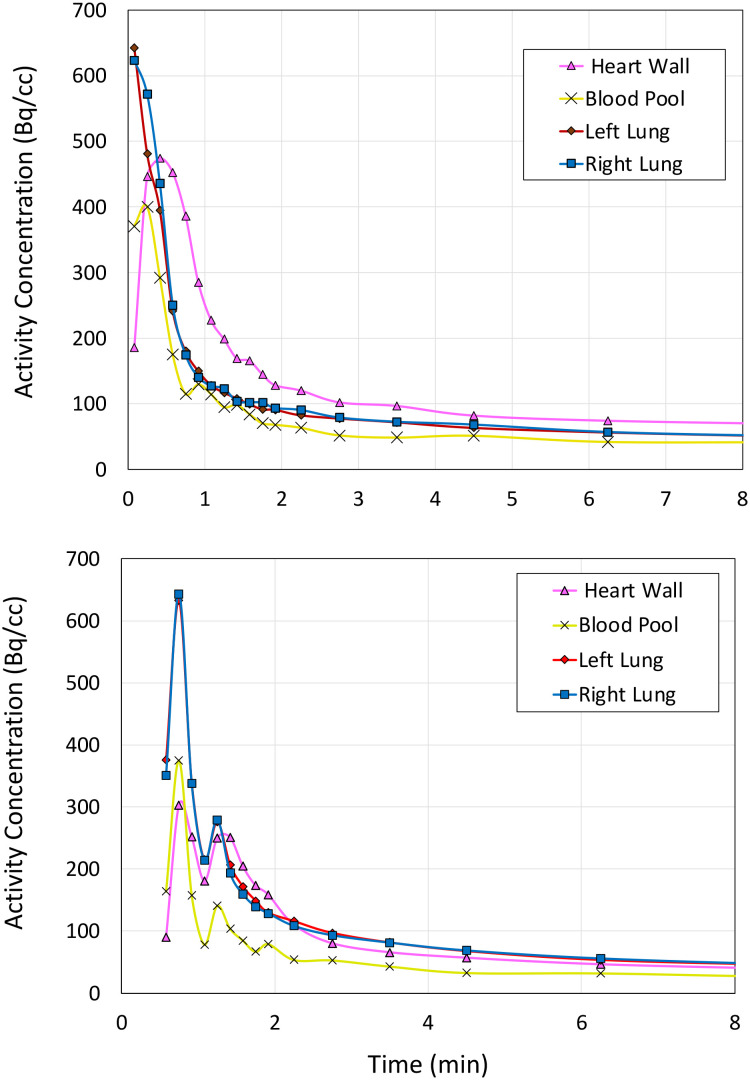
Time activity curves displaying kinetic data of rodent baseline control study (upper) and **1-H** blocking study (lower) (see ESI† for further rodent and NHP PET data).

**Scheme 3:**
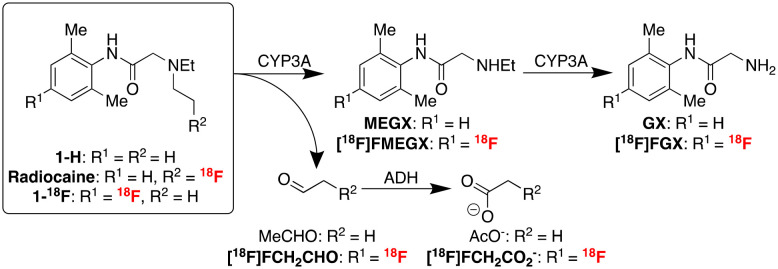
Known metabolic pathway for lidocaine **1-H** and analogous metabolic pathways predicted for radiocaine and **1-**^**18**^**F**.

We next imaged Sprague–Dawley rodents using **1-**^**18**^**F** (Fig. 2 and 3). To rationalise the binding and clearance profiles of **1-**^**18**^**F** and radiocaine, we considered the probable metabolic pathways of each imaging agent to predict the fate of each fluorine-18 label based on the known metabolism of **1-H** (Scheme 3). Critically, **1-H** is rapidly distributed *in vivo* with an initial biological half-life of 30 min. Clearance occurs at a rate of 1.44 L min^−1^, and >95% of the parent compound is converted to various metabolites, including monoethylglycinexylidide (**MEGX**) and glycinexylidide (**GX**), by CYP. Deethylation produces acetaldehyde, which can undergo oxidation by alcohol dehydrogenase to form acetate, which is converted into acetyl CoA for entry into the TCA cycle.^9^ In analogy, the [^18^F]fluoroethyl chain in radiocaine may be susceptible to this process, forming [^18^F]fluoroacetaldehyde ([^18^F]FCH_2_CHO) followed by [^18^F]fluoroacetate ([^18^F]FCH_2_CO_2_^−^), which would instead be trapped in the TCA cycle owing to the presence of the C–F bond. Indeed, cardiac uptake has been documented for [^18^F]fluoroacetate.^10,11^ Conversely, **1-**^**18**^**F** should produce the fluorine-18 labelled analogues of monoethylglycinexylidide and glycylxylidide, **[**^**18**^**F]FMEGX** and **[**^**18**^**F]FGX**, respectively.

**Fig. 4:**
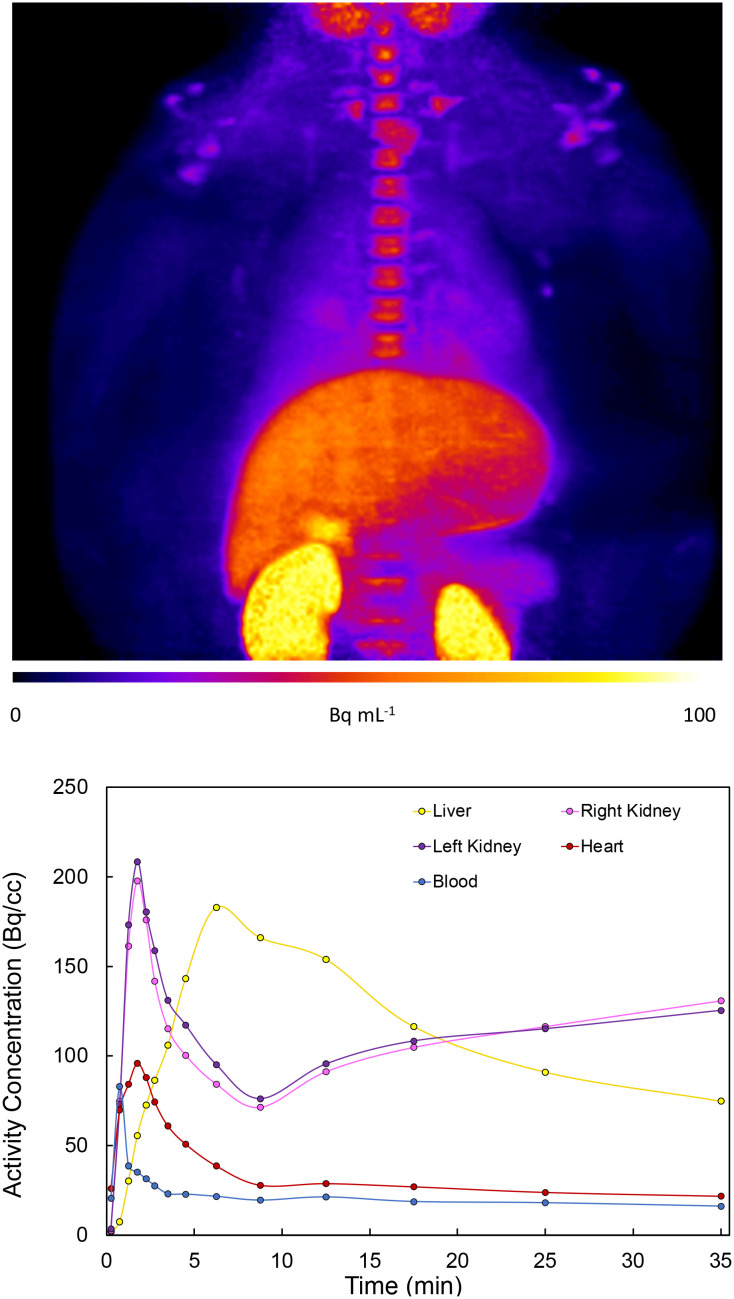
Upper: Non-human primate total summed image frames with fire table maximum intensity projection (baseline control study). Lower: Time activity curves displaying kinetic data of NHP baseline control study. No blocking study was performed owing to low cardiac uptake in the baseline study (see ESI† for further rodent and NHP PET data).

The rodent PET data shows that **1-**^**18**^**F** indeed exhibits uptake in the myocardium (4% ID g^−1^, Fig. 2) followed by rapid elimination, with most of the signal detected within 15 min from the start of the scan. Lung uptake was also recorded, which is consistent with the pharmacokinetics of **1-H**.^12^ For cardiothoracic regions of interest (*e.g.*, heart wall and lungs), **1-**^**18**^**F** has fast clearance (*ca.* 2 min), whereas the liver, kidneys, and bladder show either much slower clearance (>90 min in the liver, see ESI† for further time activity curves) or retention. However, an experimentally significant difference exists between the baseline and lidocaine-blocking images. Therefore, to account for the possible differences in metabolic rates and mechanisms between species, primate PET imaging studies were subsequently conducted using **1-**^**18**^**F** (Fig. 4). Very rapid clearance of **1-**^**18**^**F** was observed from the heart (*ca.* 2 min post-injection) and a significant uptake in the kidneys and bladder, which is consistent with the generation and renal uptake of metabolites such as **[**^**18**^**F]FMEGX** and **[**^**18**^**F]FGX** (see ESI† for further rodent and NHP PET data). Compared to radiocaine, this low myocardial uptake may be explained by the absence of the labelled metabolite [^18^F]fluoroacetate, which likely confounds Na_V_1.5 occupancy evaluation *via* PET. Therefore, the apparent retention of radiocaine may not originate from Na_V_1.5 binding alone. Combined, these preliminary observations call into question the Na channel target specificity of radiocaine and **1-**^**18**^**F** and their suitability as agents for assessing Na_V_1.5 occupancy.

## Conclusion

A newly optimised and automated preparation of **1-**^**18**^**F**, an analogue of the anaesthetic lidocaine **1-H** containing an aromatic C–^18^F bond, is described. This protocol leverages sequential Ir C–H borylation/Cu-mediated radiofluorination for rapid labelling beginning with lidocaine **1-H**. Subsequently, **1-**^**18**^**F** was employed in preclinical rodent and non-human primate studies to gain insight into the metabolic properties of fluorine-18 labelled amino amide anaesthetic derivatives. In principle, these agents (*e.g.*, radiocaine, **1-**^**18**^**F**) could support the discovery of cardiovascular therapeutics and the evaluation of disease progression associated with myocardial voltage-gated Na channels. However, our imaging experiments provide evidence that fluorine-18 labelled amino amide anaesthetics based on the lidocaine scaffold are unsuitable for imaging these channels, likely owing to rapid metabolism. In particular, the metabolism of **1-**^**18**^**F** may generate fluorine-18 labelled analogues of **MEGX** and **GX***via* dealkylation, leading to rapid renal uptake/clearance and poor myocardial uptake. Radiocaine, which likely targets Na_V_1.5 initially, can similarly undergo rapid oxidative dealkylation. This may facilitate the myocardial accumulation of [^18^F]fluoroacetate, an agent known to exhibit cardiac retention. Since fluoroacetate is trapped in the TCA cycle, the *in vivo* myocardial PET signal observed preclinically using radiocaine may not entirely be due to specific Na_V_1.5 binding since [^18^F]fluoroacetate cannot evaluate channel occupancy. Therefore, future studies should further evaluate the pharmacokinetic changes accompanying fluorine installation into the lidocaine scaffold, including at the ethyl chains. For example, studies that quantify the Na_V_1.5 occupancy properties of ^19^F-radiocaine would provide further insights into imaging efficacy. However, it is noteworthy that ^19^F-fluorinated analogues of **1-H** exhibit reduced potency, which could further complicate the development of efficacious Na channel imaging agents based on **1-H**.^13^ Reflecting these challenges, upcoming research in our laboratory shall pursue new scaffolds for developing cardiac imaging agents, including quantifying binding affinity and selectivity of more efficacious SCN5A PET imaging agents toward Na_v_1.5 and other subfamilies. For example, Na ion channel blockers from classes Ia and Ic that exhibit different channel association/dissociation properties and metabolic pathways may show promise.

## Ethical statement

Non-human primate and rodent PET imaging studies were conducted under the supervision of the University of Michigan, USA, and its Institutional Animal Care and Use Committee (IACUC approval number PRO00011715) according to approved protocols and all applicable federal, state, local, and institutional laws or guidelines governing animal research.

## Data availability

The data supporting this article have been included as part of the ESI.†

## Conflicts of interest

The authors declare that they have no known competing financial interests or personal relationships that could influence the research reported in this paper.

## Supplementary Material

MD-OLF-D4MD00293H-s001

## References

[cit1] Bennett D. L., Clark A. J., Huang J., Waxman S. G., Dib-Hajj S. D. (2019). Physiol. Rev..

[cit2] Li Z.-M., Chen L.-X., Li H. (2019). Curr. Med. Sci..

[cit3] Feldman H. S., Hartvig P., Wilkund L., Doucette A. M., Antoni G., Gee A., Ulin J., Langstrom B. (1997). Biopharm. Drug Dispos..

[cit4] Hooker J. M., Strebl M. G., Schroeder F. A., Wey H.-Y., Ambardekar A. V., McKinsey T. A., Schoenberger M. (2017). Sci. Rep..

[cit5] Wright J. S., Sharninghausen L. S., Preshlock S., Brooks A. F., Sanford M. S., Scott P. J. H. (2021). J. Am. Chem. Soc..

[cit6] Preshlock S. M., Plattner D. L., Maligres P. E., Krska S. W., Maleczka Jr. R. E., Smith III M. R. (2013). Angew. Chem., Int. Ed..

[cit7] Larsen M. A., Hartwig J. F. (2014). J. Am. Chem. Soc..

[cit8] Zischler J., Kolks N., Modemann D., Neumaier B., Zlatopolskiy B. D. (2017). Chem. – Eur. J..

[cit9] Hollunger G. (1961). Basic Clin. Pharmacol. Toxicol..

[cit10] Ho C.-L., Cheung M.-K., Chen S., Cheung T. T., Leung Y. L., Cheng K. C., Yeung W. D. (2012). Mol. Imaging.

[cit11] Ponde D. E., Dence C. S., Oyama N., Kim J., Tai Y.-C., Laforest R., Siegel B. A., Welch M. J. (2007). J. Nucl. Med..

[cit12] Orlando R., Piccoli P., De Martin S., Padrini R., Floreani M., Palatini P. (2004). Clin. Pharmacol. Ther..

[cit13] Löfgren N., Lundqvist B., Lindström S. (1955). Acta Chem. Scand..

