# Supplementary material for: Automated radiosynthesis and preclinical imaging of a novel [18F]fluorolidocaine analogue via sequential C–H radiolabelling
Source: RSC Med Chem. 2024 Jul 31;15(9):3223–7. doi: 10.1039/d4md00293h (PMC11339636; doi:10.1039/d4md00293h)
Supplement: MD-015-D4MD00293H-s001 [file MD-015-D4MD00293H-s001.pdf]

Supplementary Information (SI) for RSC Medicinal Chemistry.
This journal is © The Royal Society of Chemistry 2024

Automated Radiosynthesis and Preclinical Imaging of a Novel [^{18}F]Fluorolidocaine Analogue via Sequential C-H Radiolabelling

Madison Frazier[a†], Jay S. Wright*[a†], David Raffel[a], Jenelle Stauff[a], Wade Winton[a], Peter J. H. Scott*[a], and Allen F. Brooks*[a]

1. General

1.1 Materials and Methods

Unless otherwise stated, all chemicals were synthesised or obtained commercially. Anhydrous solvents, including dimethylacetamide (DMA), tetrahydrofuran (THF), 2-methyltetrahydrofuran (2-MeTHF), fitted with a Sure/Seal™, ChemSeal™, or AcroSeal™ septum, were obtained from Millipore Sigma or Fisher Scientific and used as received. Sterile dose vials were purchased from Hollister-Stier. QMA-light (WAT023525) and Sep-Pak C18 1 cc Vac 50 mg Cartridges (186000308) were purchased from Waters Corporation. QMA-light Sep-Paks were flushed with 10 mL of EtOH, 10 mL of aqueous 0.5M KOTf, and 10 mL of milli-Q H_2O before use. Sep-Pak C18 1 cc Vac 50 mg cartridges were flushed with 10 mL EtOH and 10 mL H_2O before use. $[Ir(cod)(OMe)]_2$ was synthesised starting from $IrCl_3$ hydrate.[1]

1.2 Instrument Information

NMR spectra were obtained at 25 °C on a Varian MR400 (401 MHz for ^{1}H; 376 MHz for ^{19}F; 123 MHz for ^{13}C) or Varian VNMRS 500 (500.09 MHz for ^{1}H; 470.56 MHz for ^{19}F; 125.75 MHz for ^{13}C) spectrometer. ^{1}H and ^{13}C NMR chemical shifts are reported in parts per million (ppm), with the residual solvent peak used as an internal reference. ^{1}H and ^{19}F NMR multiplicities are reported as follows: singlet (s), doublet (d), triplet (t), quartet (q), quintet (quin), and multiplet (m). Compounds were characterised via NMR spectroscopy, high-resolution mass spectrometry, and/or analytical high-performance liquid chromatography. Mass spectral data were obtained on a Micromass Magnetic Sector Mass Spectrometer in electrospray (ESI) ionisation mode or Micromass AutoSpec Ultima Magnetic Sector Mass Spectrometer in electron ionisation m/z (EI) mode. Automated flash chromatography was conducted on a Biotage Isolera Prime or One system equipped with a KP-Sfär cartridge (10-50 g). Analytical HPLC analysis was performed using a Shimadzu LC-20AD/T LPGEKIT system fitted with a CBM-20A controller, SIL 20AHT injector, SPD 20A UV detector, DGU 20A SR degasser, and CTO-20A column oven with an in-series B-FC-3300 radiation detector for observing gamma emissions.

2. Chemistry

2.1 Non-radioactive Chemistry

Lidocaine (**1-H**) was investigated for sequential C-H radiolabelling under a modified protocol reported by our laboratories previously that afforded [^{18}F]fluorolidocaine in 15 ± 8% radiochemical conversion under manual conditions (n=3).[2] Attempts to radiosynthesise [^{18}F]fluorolidocaine from this crude Ir borylation mixture under automated conditions were unsuccessful, and this was attributed to the sequestration of HBpin via rapid N-H borylation to form adduct **2-Bpin**, resulting in insufficient C-borylated products for labelling. Although **2-Bpin** is hydrolytically unstable, as demonstrated by quenching experiments, crude ^{1}H NMR evidence for its formation is presented.

2.1.1 Ir Borylation of Lidocaine Conditions A

- <u>6-(2,6-Dimethylphenyl)-9,9-diethyl-2,2,3,3-tetramethyl-1,4-dioxa-6,9λ^4-diaza-5λ^4-boraspiro[4.4]nonan-7-one **(2-Bpin, crude)**</u>
- <u>2-(Diethylamino)-*N*-(2,6-dimethyl-4-(4,4,5,5-tetramethyl-1,3,2-dioxaborolan-2-yl)phenyl)-*N*-(4,4,5,5-tetramethyl-1,3,2-dioxaborolan-2-yl)acetamide **(3-(Bpin)$_2$, crude)**</u>
- <u>2-(Diethylamino)-*N*-(2,6-dimethyl-4-(4,4,5,5-tetramethyl-1,3,2-dioxaborolan-2-yl)phenyl)acetamide **(3-Bpin, crude)**</u>

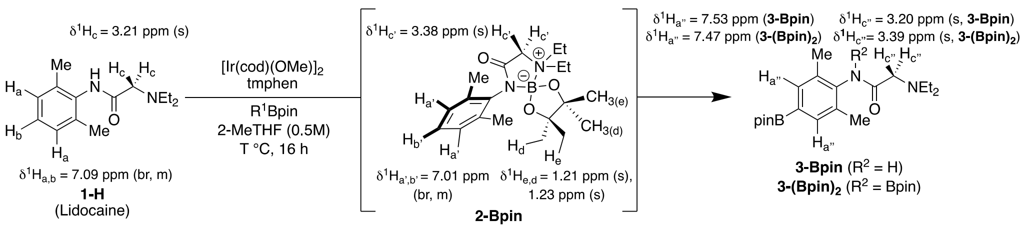

An oven-dried reaction tube was charged with $[Ir(cod)(OMe)]_2$ (3.3 mg, 1.0 mol%) and 3,4,7,8-tetramethyl-1,10-phenanthroline (2.4 mg, 2.0 mol%) and subject to three high vacuum purge/Ar refill cycles. Degassed 2-MeTHF (0.5 mL) followed by pinacolborane (0.14 mL, 0.9 mmol, 1.8 equiv.) was added, and the tube was sealed. The resulting dark solution was stirred rapidly at 80 °C for 2 min before adding a solution of **1-H** (117 mg, 0.5 mmol, 1.0 equiv.) in degassed 2-MeTHF (0.5 mL). The reaction was stirred at 80 °C for 16 h. Significant precipitation was noted over the course of the reaction. Reactions that were not preheated before substrate addition did not proceed. A heterogeneous 100 µL aliquot was dried in a Schlenk tube, dissolved in anhydrous $CDCl_3$, and transferred under Schlenk conditions to a purged/Ar-refilled septa-cap NMR tube. ^1H NMR spectroscopy analysis was conducted using 1,3,5-trimethoxybenzene as an internal standard. Three new products were observed and tentatively assigned as **2-Bpin** (24% NMR yield), **3-Bpin** (15% NMR yield), and **3-(Bpin)$_2$** (27%). Evidence for these assignments is discussed and presented below. The generation of these three new compounds was reproducible, although the distribution could somewhat vary run-to-run. This likely originates from adventitious N-B hydrolysis. **1-H** was also observed in 9% NMR yield, and we suggest that the mild reaction heterogeneity explains the apparent loss of material based on these analyses.

Discussion of Product Generation in the Borylation of 1-H Under Conditions A: Aromatic Ir borylation induces a diagnostic downfield shield in *ortho*-protons within approximately 0.5 ppm. Other *ortho*-nuclei are similarly deshielded to varying degrees. From the crude ^1H NMR spectrum, one of the new products was generated with an aromatic signal instead with an *upfield* shift at 7.01 ppm relative to **1-H** (7.08 ppm). N-H borylation is known to precede C-H borylation and form the corresponding N-boryl adducts, so we suggest that the amide N-H of **1-H** first undergoes borylation. Significant downfield shifts of the remaining proton environments proximal to the amide nitrogen were also observed relative to **1-H**, and we suggest that cyclisation of the tertiary amine into the p-orbital of the boron atom causes this via charge build-up and enforcement of conformational rigidity. One of the largest shifts can be seen at the 4H ethyl methylene unit H_c. In **1-H**, this group shifts to 2.69 ppm, whilst in **2-Bpin** and **3-(Bpin)$_2$**, which likely contain an N-B ylide, this group shifts to 3.14 ppm and 3.24 ppm, respectively. Furthermore, these signals appear as a quartet in **1-H** but are altered to overlapping broad lines in the crude ^1H NMR spectrum. These significant downfield shifts and broadenings are consistent with the build-up of cationic charge at the tertiary amine and conformational rigidity that may feature in a cyclised N-B adduct. See Table 1 for similar examples of this phenomenon. Based on these observations, we suggest that **2-Bpin** is generated in 24% NMR yield. Attempts to further analyse **2-Bpin** and **3-(Bpin)$_2$** were unsuccessful, primarily owing to the poor hydrolytic stability of many N-B bonds, so these analyses do not constitute a formal and unambiguous product characterisation. A summary of the major ^1H NMR shift differences is represented in Table 1. The two other products generated in the borylation of **1-H** have aromatic signals downfield within 0.5 ppm relative to **1-H**, consistent with the deshielding effect that the aryl boronate substituent exerts on the *ortho*-protons. This suggests that these compounds are derivatives of **1-H** substituted with a Bpin group at H_b since the C-H cleavage is sterically controlled. One of the aromatic lines has remaining signals with shifts comparable to **2-Bpin**, whilst the other has remaining shifts comparable to **1-H**. Therefore, we suggest that the remaining two species correspond to mono-borylated **3-Bpin** and diborylated **3-(Bpin)$_2$**. It is likely that **3-(Bpin)$_2$** is also intramolecularly cyclised in analogy to **2-Bpin**.

/ppm	H_a/H_b	$ArCH_3$	NH	H_c	NCH_2CH_3	NCH_2CH_3	H_d/H_e

**1-H**	7.09 (br)	2.23 (s)	8.92 (br, s)	3.21 (s)	2.69 (q, *J* = 7.1 Hz)	1.14 (t, *J* = 7.1 Hz)	-
**2-Bpin**	7.01 (br)	2.30 (s)	-	3.38 (s)	3.14 (br, overlaps)	-	1.21 (s), 1.23 (s)
**3-Bpin**	7.53 (s)	2.22 (s, overlaps)	8.95 (br, s)	3.20 (s)	2.69 (q, *J* = 7.1 Hz, overlaps)	1.13 (t, overlaps)	-
**3-(Bpin)$_2$**	7.47 (s)	2.31 (s)	-	3.39 (s)	3.24 (br, overlaps)	-	-

Table 1: ^{1}H NMR Shifts of Lidocaine and Putatively Assigned Products from the Crude Reaction Mixture Obtained in the Borylation of **1-H** under Conditions A. Signals that are not described are either not present or not accurately discernible.

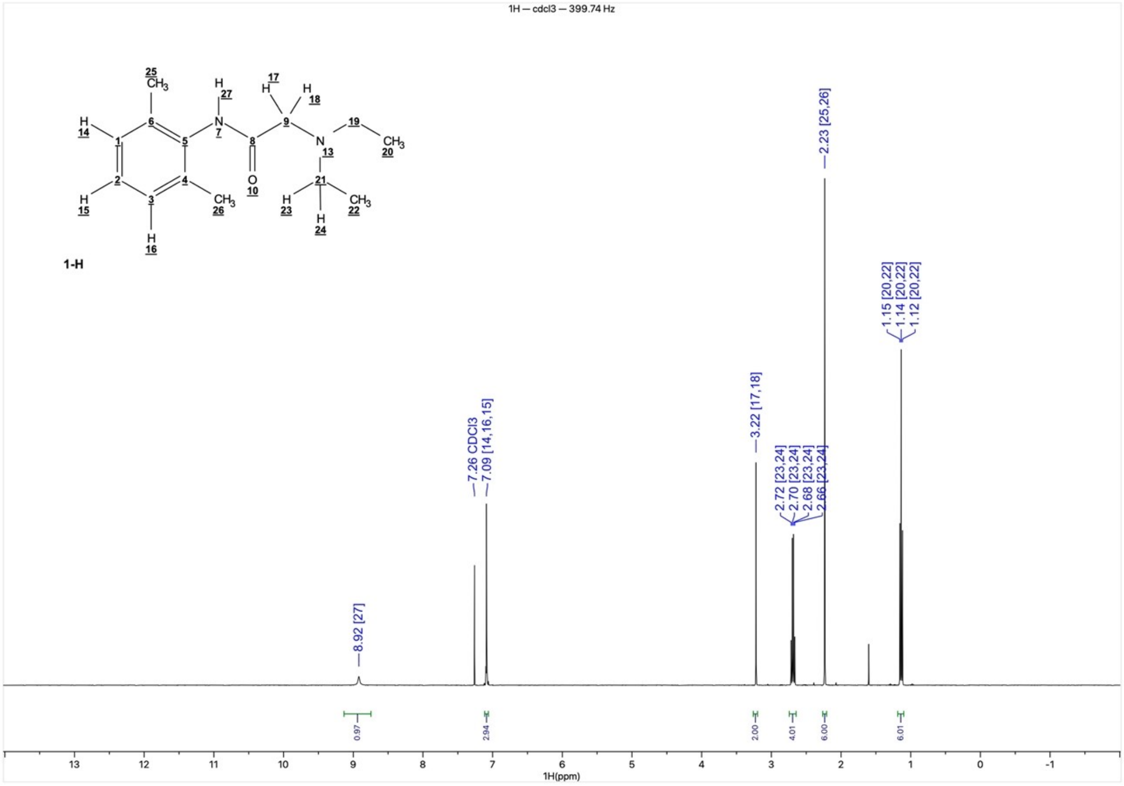

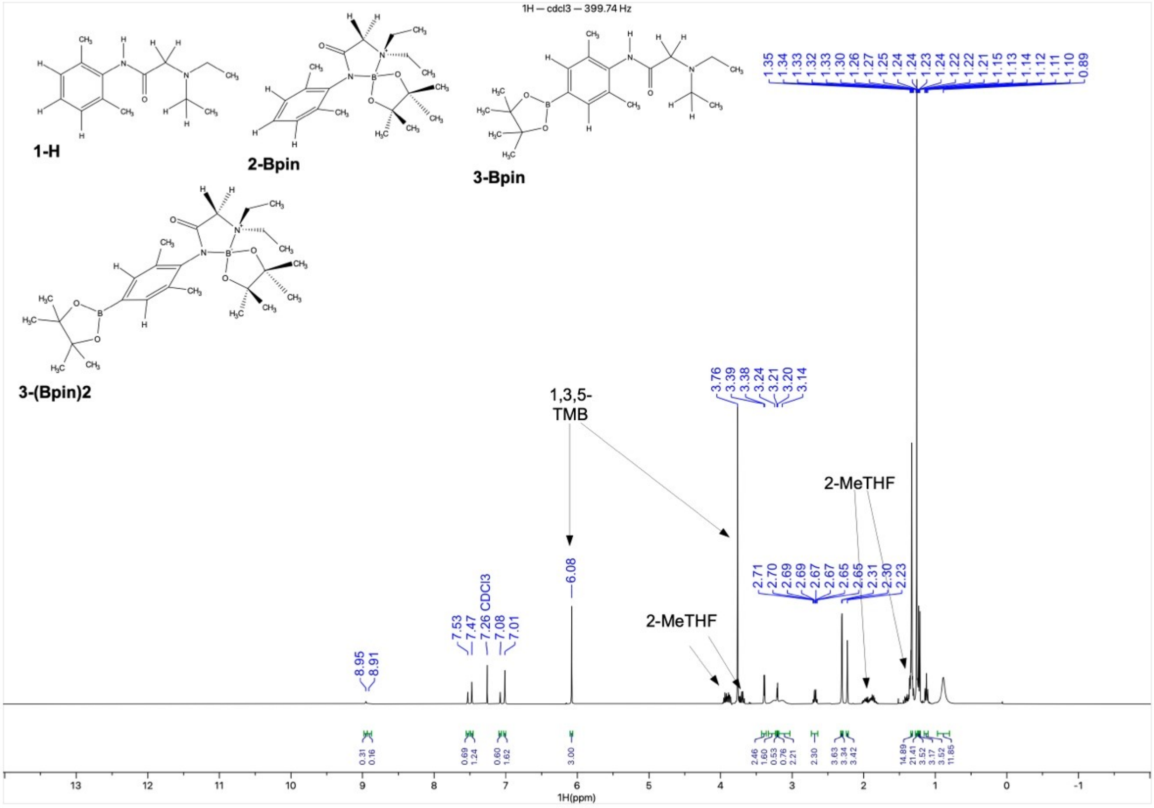

Figure 1: ^{1}H NMR Spectrum of Commercial Lidocaine (**1-H**) Sample (Upper). ^{1}H NMR Spectrum of Crude Ir Borylation of **1-H** under Conditions A (Lower).

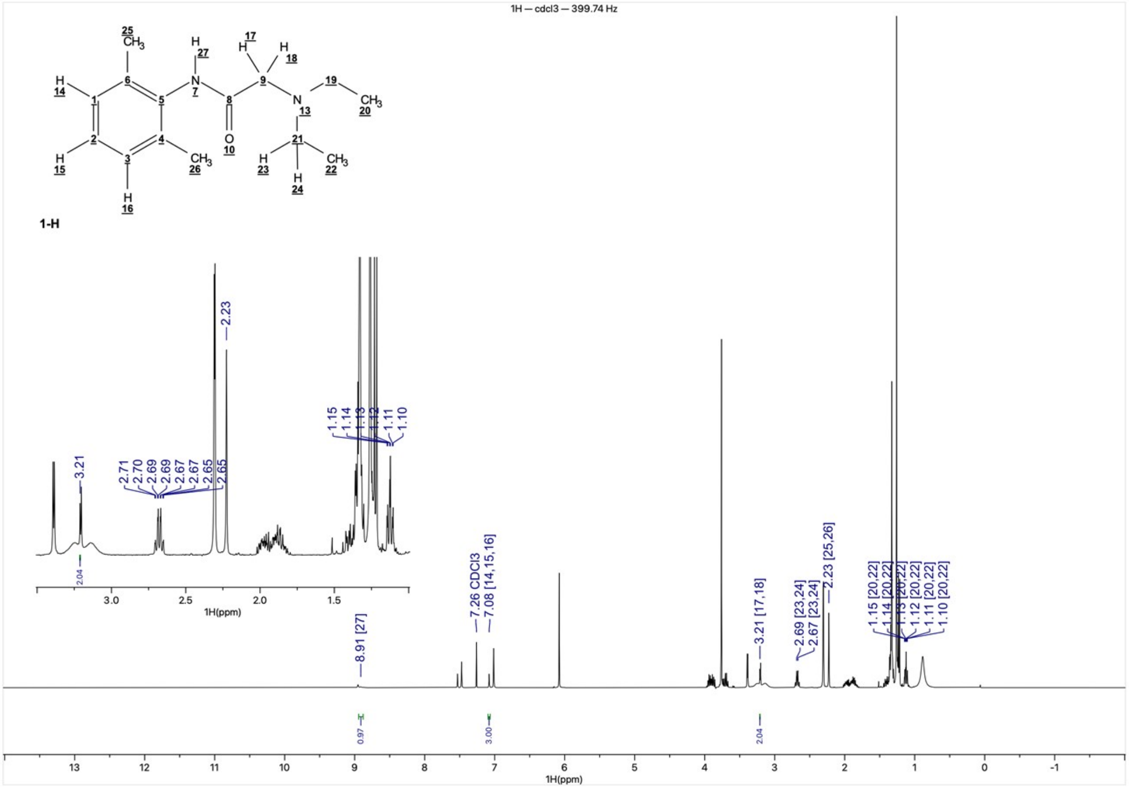

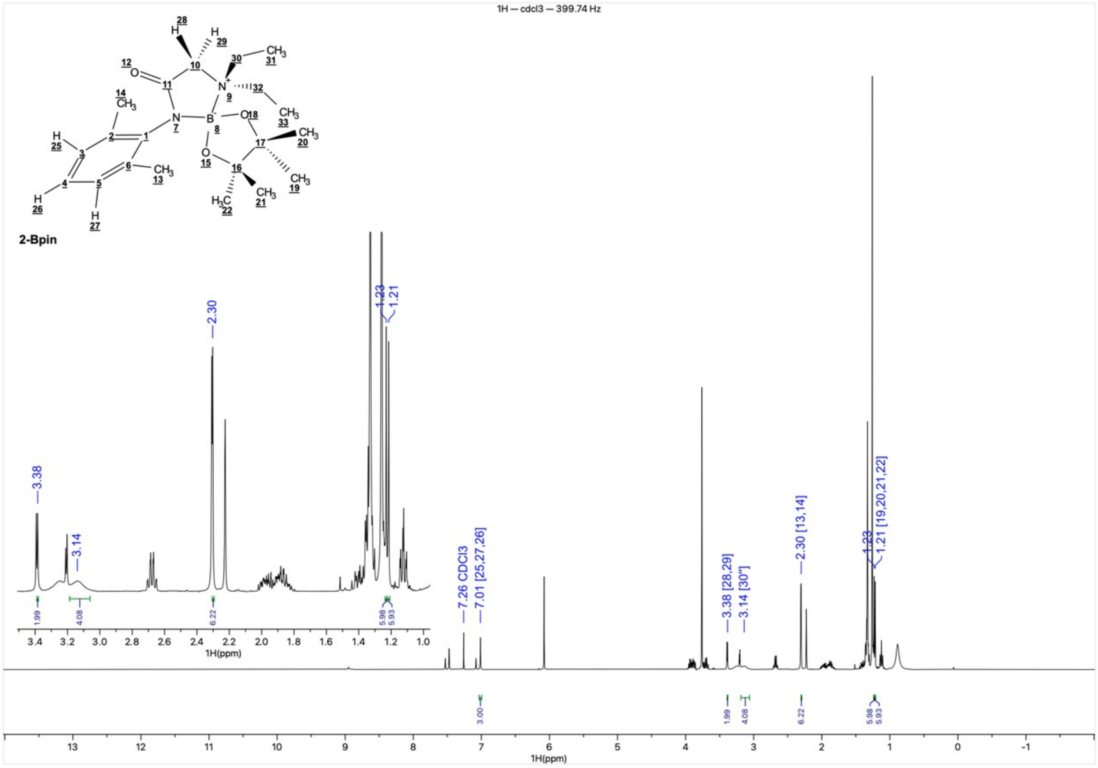

Figure 2: Crude ^{1}H NMR Spectrum Showing Ir Borylation of **1-H** Under Conditions A. Peak Pickings Correspond to Putative Assignments for **1-H** (Upper) and **2-Bpin** (Lower).

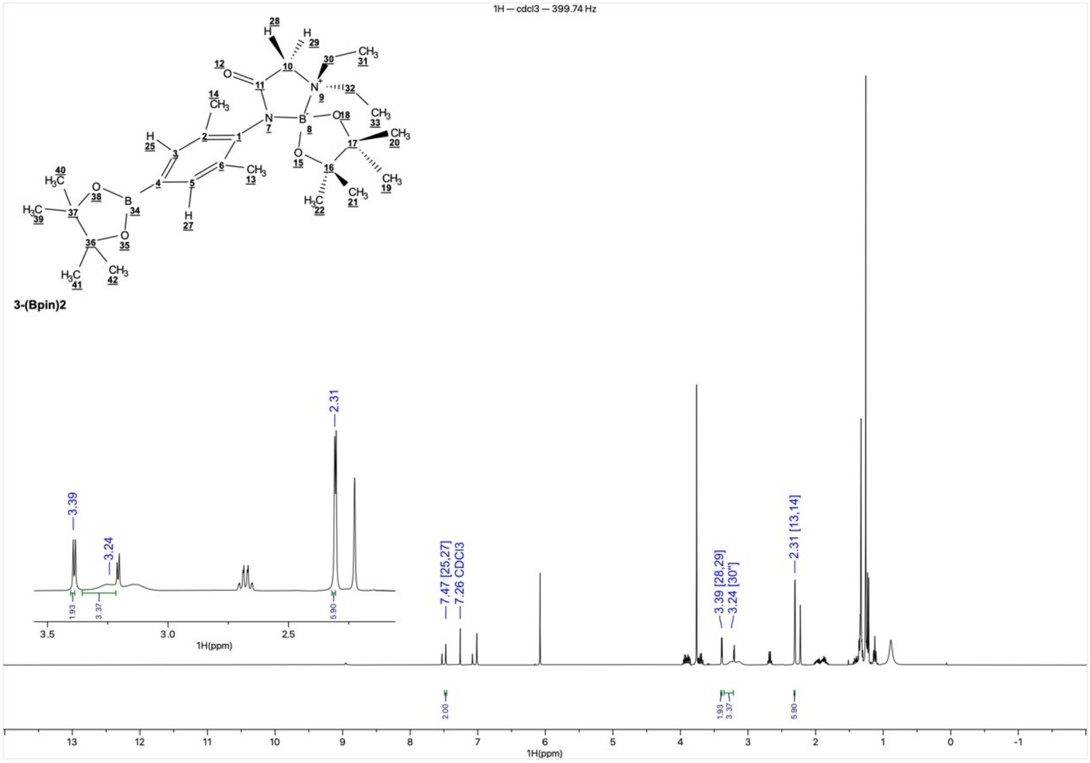

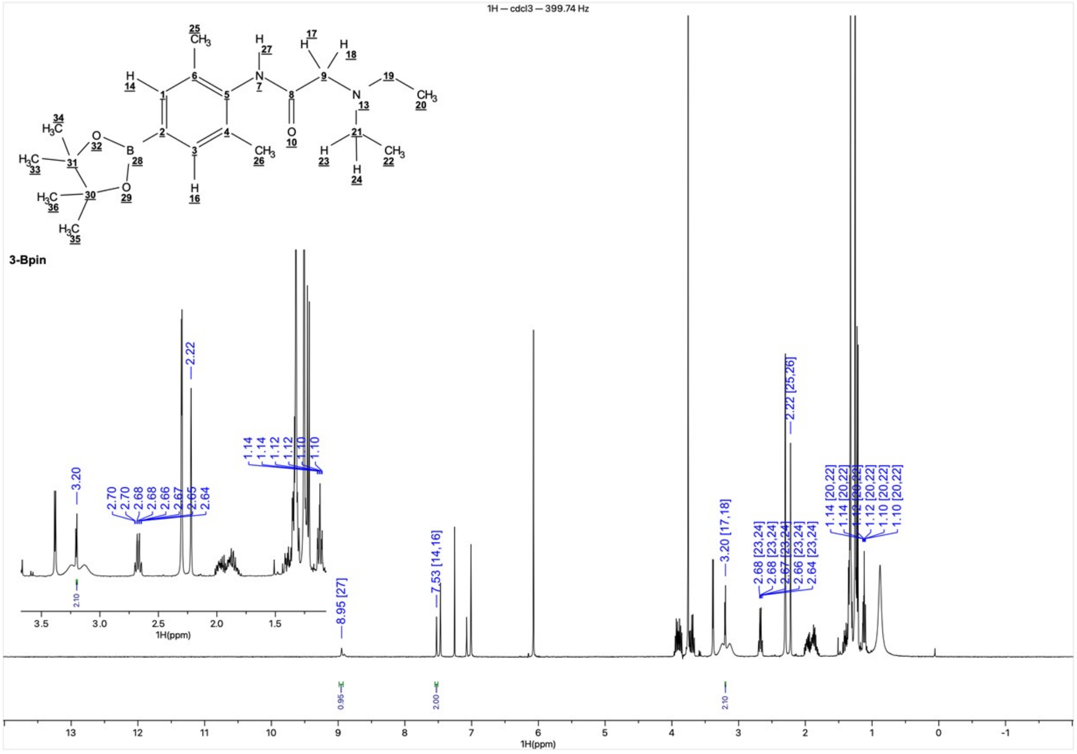

Figure 3: Crude ^{1}H NMR Spectrum Showing Ir Borylation of **1-H** Under Conditions A. Peak Pickings Correspond to Putative Assignments for **3-(Bpin)$_2$** (Upper) and **3-Bpin** (Lower).

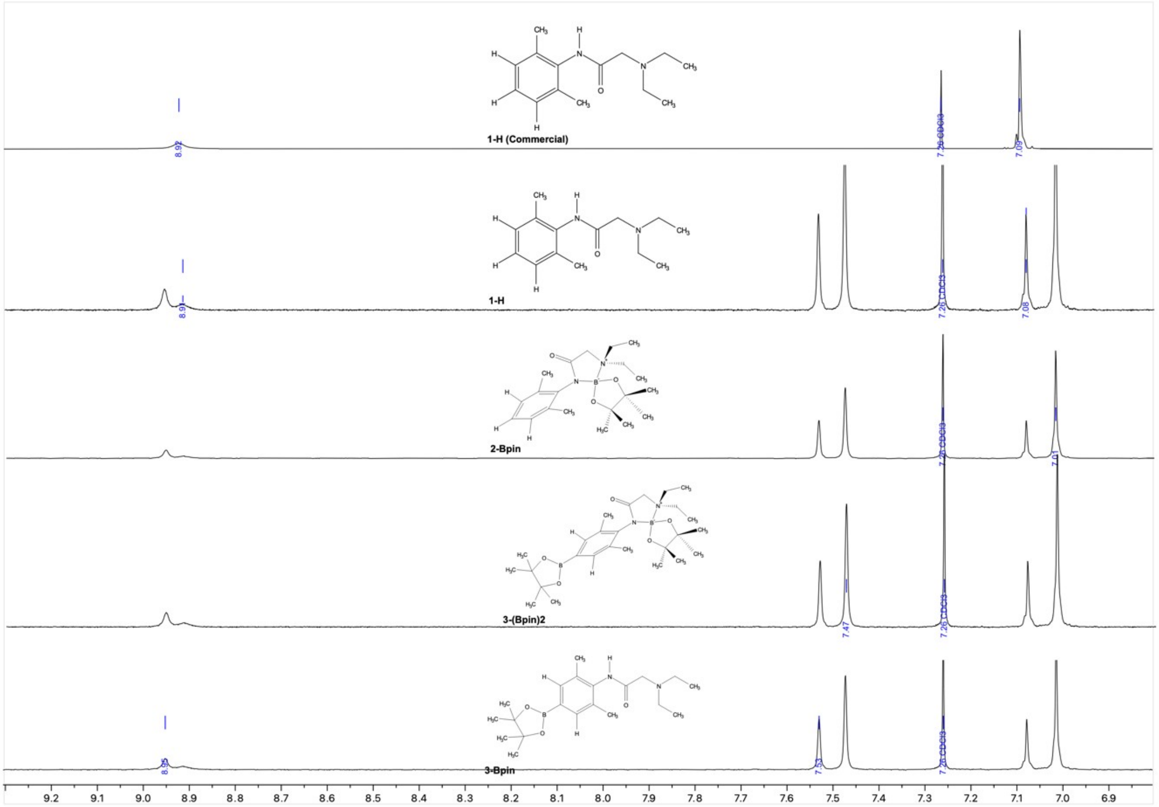

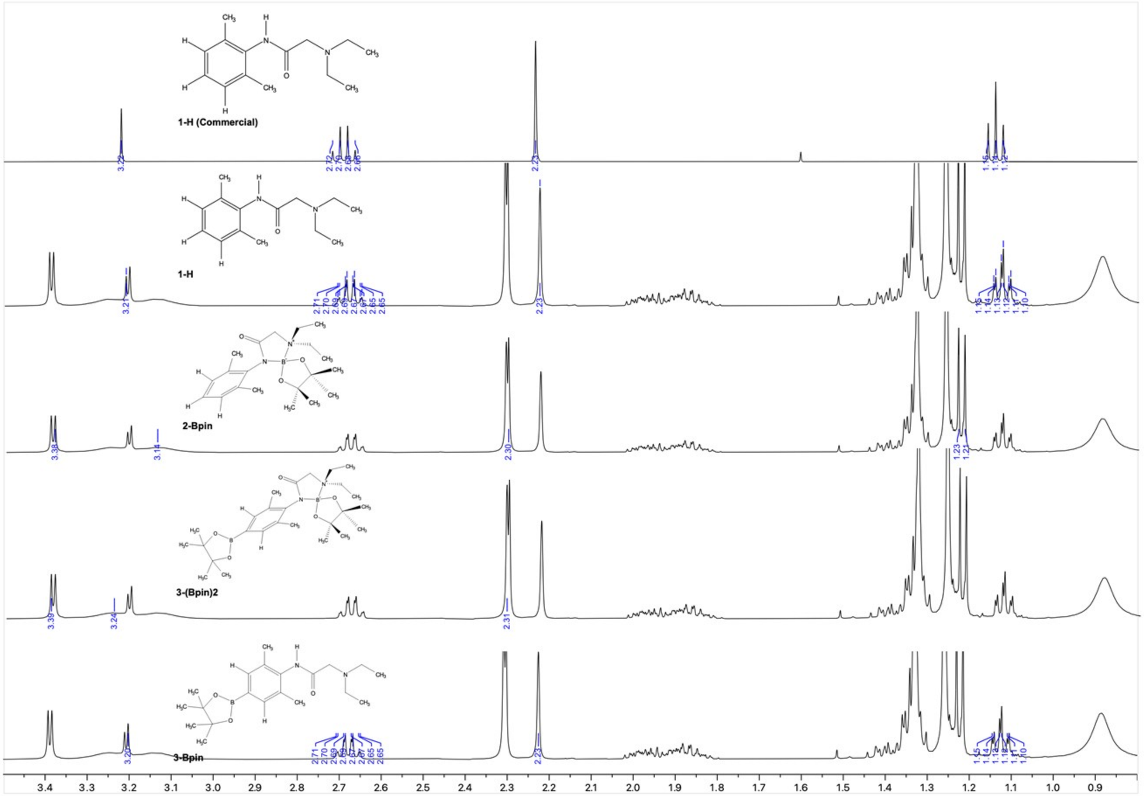

Figure 4: ^{1}H NMR Spectra of Commercial **1-H** and crude Ir Borylation Reaction of **1-H** Under Conditions A. Both stacks show a ^{1}H NMR Spectrum of a commercial sample of **1-H**, followed by the same crude Ir borylation reaction mixture with peak pickings that correspond to putative assignments. ^{1}H NMR High ppm Shifts (Upper) and Low ppm Shifts (Lower) are shown.

2.1.2 Ir Borylation of Lidocaine Conditions B

- 2-(Diethylamino)-*N*-(2,6-dimethyl-4-(4,4,5,5-tetramethyl-1,3,2-dioxaborolan-2-yl)phenyl)-*N*-(4,4,5,5-tetramethyl-1,3,2-dioxaborolan-2-yl)acetamide **(3-(Bpin)$_2$, crude)**
- 2-(Diethylamino)-*N*-(2,6-dimethyl-4-(4,4,5,5-tetramethyl-1,3,2-dioxaborolan-2-yl)phenyl)acetamide **(3-Bpin, crude)**

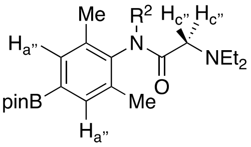

3-Bpin (R^2 = H)
3-(Bpin)$_2$ (R^2 = Bpin)

Experiment 1: An oven-dried reaction tube was charged with $[Ir(cod)(OMe)]_2$ (6.6 mg, 2.0 mol%), 3,4,7,8-tetramethyl-1,10-phenanthroline (4.8 mg, 4.0 mol%), and bis(pinacolato)diboron (279 mg, 1.1 mmol, 2.2 equiv.) and subject to three high vacuum purge/Ar refill cycles. Degassed 2-MeTHF or THF (0.5 mL) was added, and the tube was sealed. The resulting dark solution was stirred rapidly at room temperature for 2 min before adding a solution of **1-H** (117 mg, 0.5 mmol, 1.0 equiv.) in degassed 2-MeTHF or THF (0.5 mL). The reaction was stirred at 100 °C for 16 h. A 100 µL aliquot was quickly dried in air and analysed via ^{1}H NMR spectroscopy. **1-H** had been nearly completely consumed, and a new product, tentatively assigned as **3-Bpin**, was observed in approximately 95% conversion (based on starting material). Signal shifts matched **3-Bpin** tentatively generated under Conditions A (see Section 1.1.1 for assignments). Rotational isomerism was observable in this experiment in a ca. 3:1 ratio.

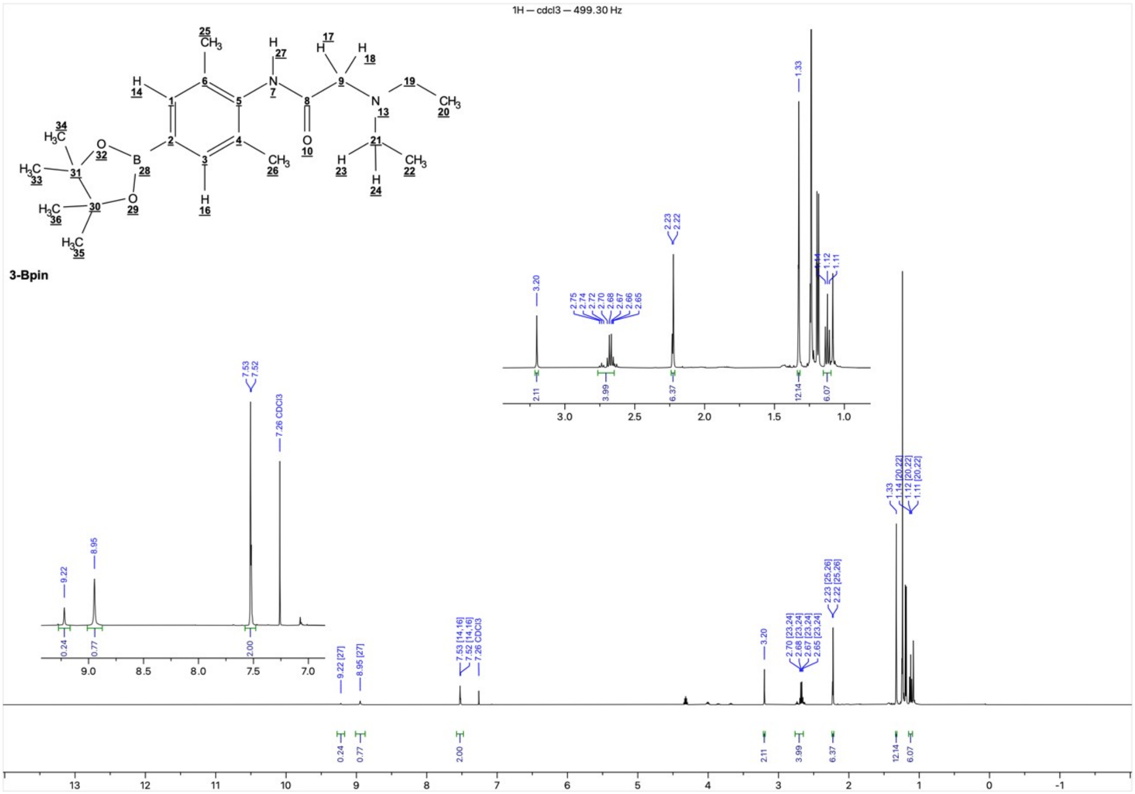

Figure 5: ^{1}H NMR Spectrum of Crude Ir Borylation of **1-H** under Conditions B, Experiment 1. Peak Pickings Correspond to Putative Assignments for **3-Bpin**.

Experiment 2: In a repeat of Experiment 1, the crude reaction mixture was transferred under Schlenk conditions to a purged/Ar-refilled septa-cap NMR tube. Analysis of this crude reaction mixture via ^{1}H NMR spectroscopy confirmed the generation of **3-Bpin** and **3-(Bpin)$_2$** in a 5:1 ratio. Treatment of this NMR sample with one drop of EtOH led to quantitative decomposition of **3-(Bpin)$_2$**, and the ^{1}H NMR spectrum showed only **3-Bpin** remaining. This is explained by rapid alcoholysis of the N-B bond in **3-(Bpin)$_2$**, converting **3-(Bpin)$_2$** to **3-Bpin**. An internal NMR standard confirmed that **3-(Bpin)$_2$** was not decomposed to other products in this experiment.

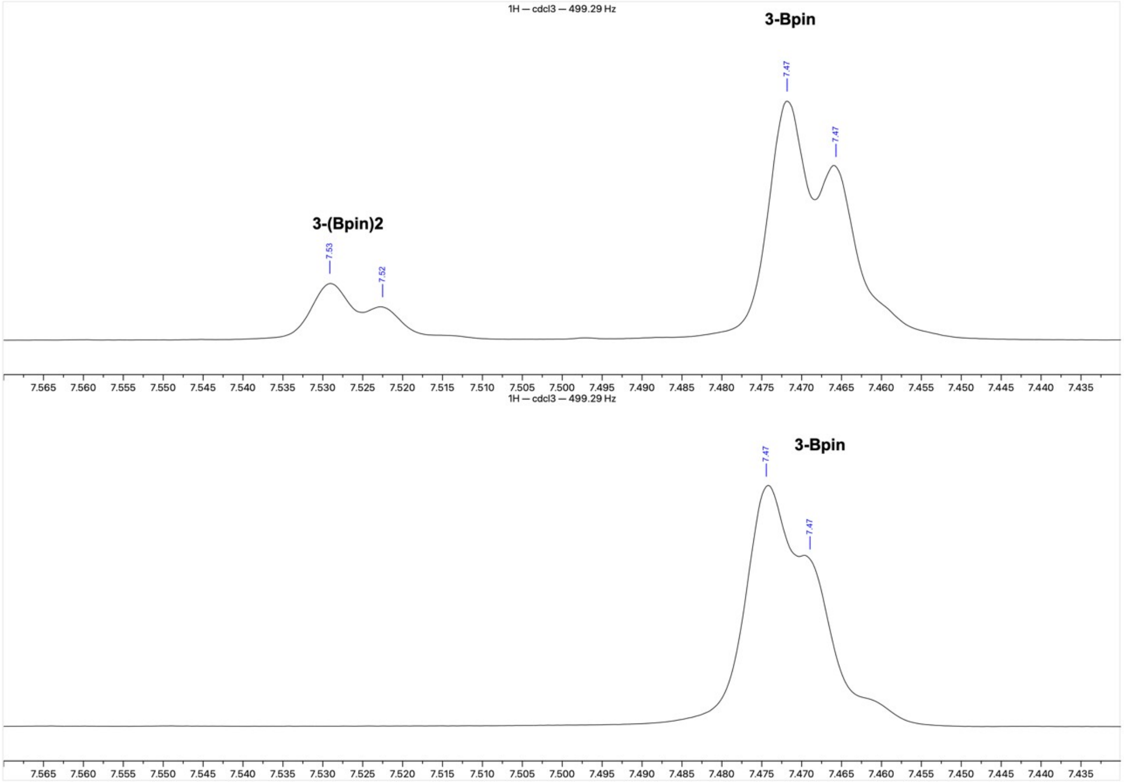

Figure 6: ^{1}H NMR Spectra of Crude Ir Borylation of **1-H** under Conditions B, Experiment 2. The Crude Reaction Mixture was Analysed under Inert Atmosphere, Showing **3-Bpin** and **3-(Bpin)$_2$** (Upper), Followed by Addition of EtOH (Lower), Leading to Rapid Decomposition of **3-(Bpin)$_2$**.

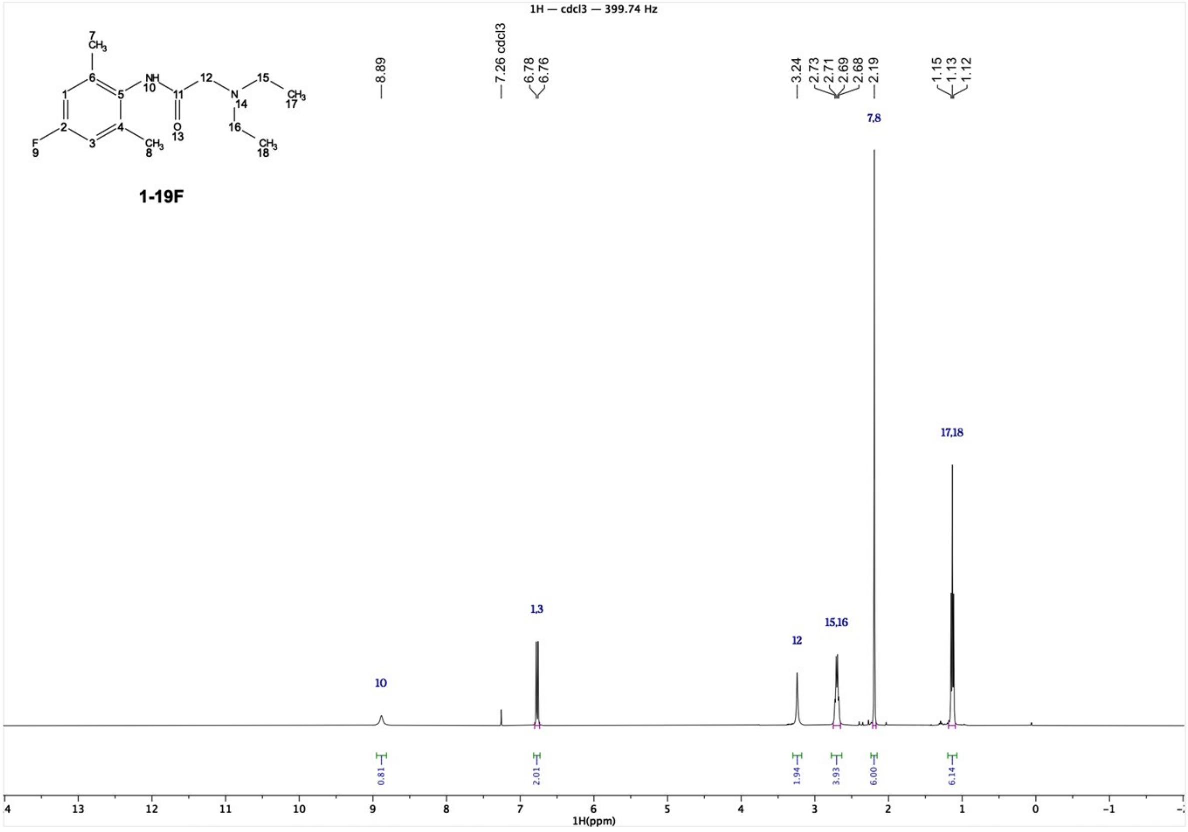

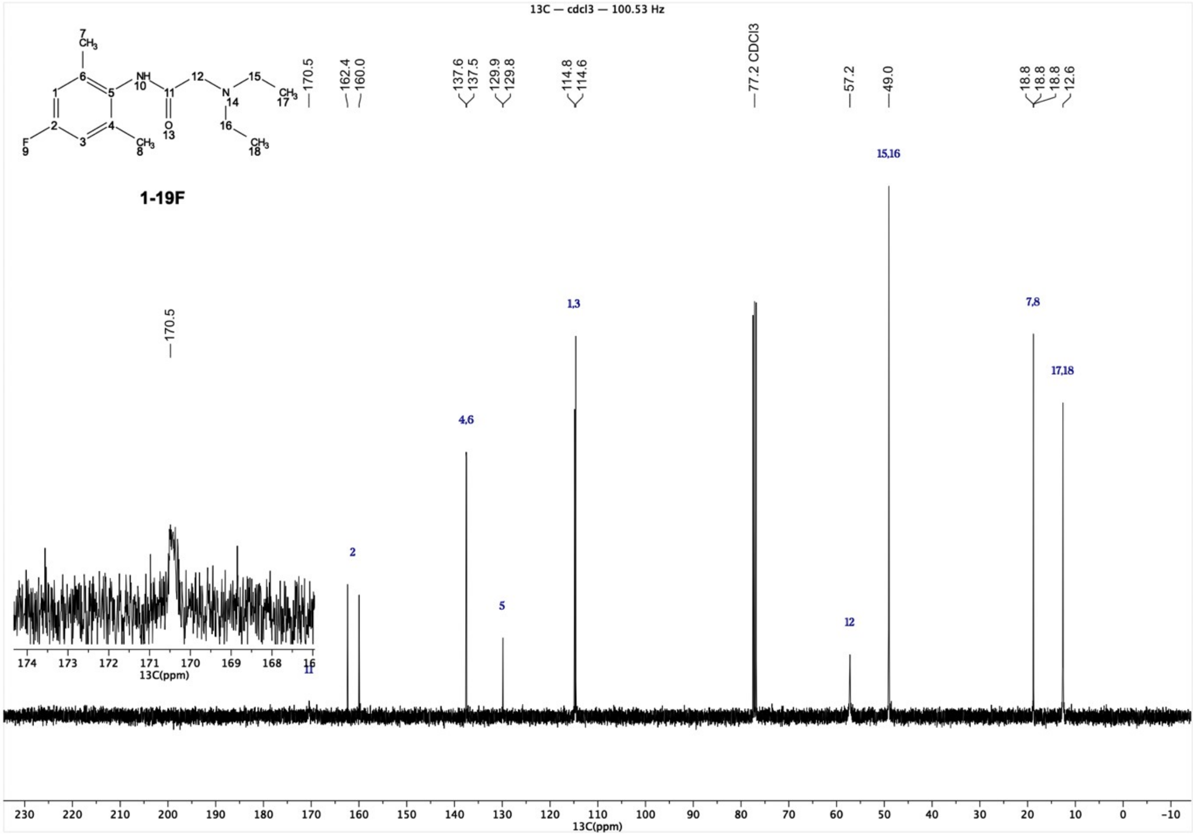

Figure 7: ^{1}H and ^{13}C NMR spectra of **1-^{19}F**

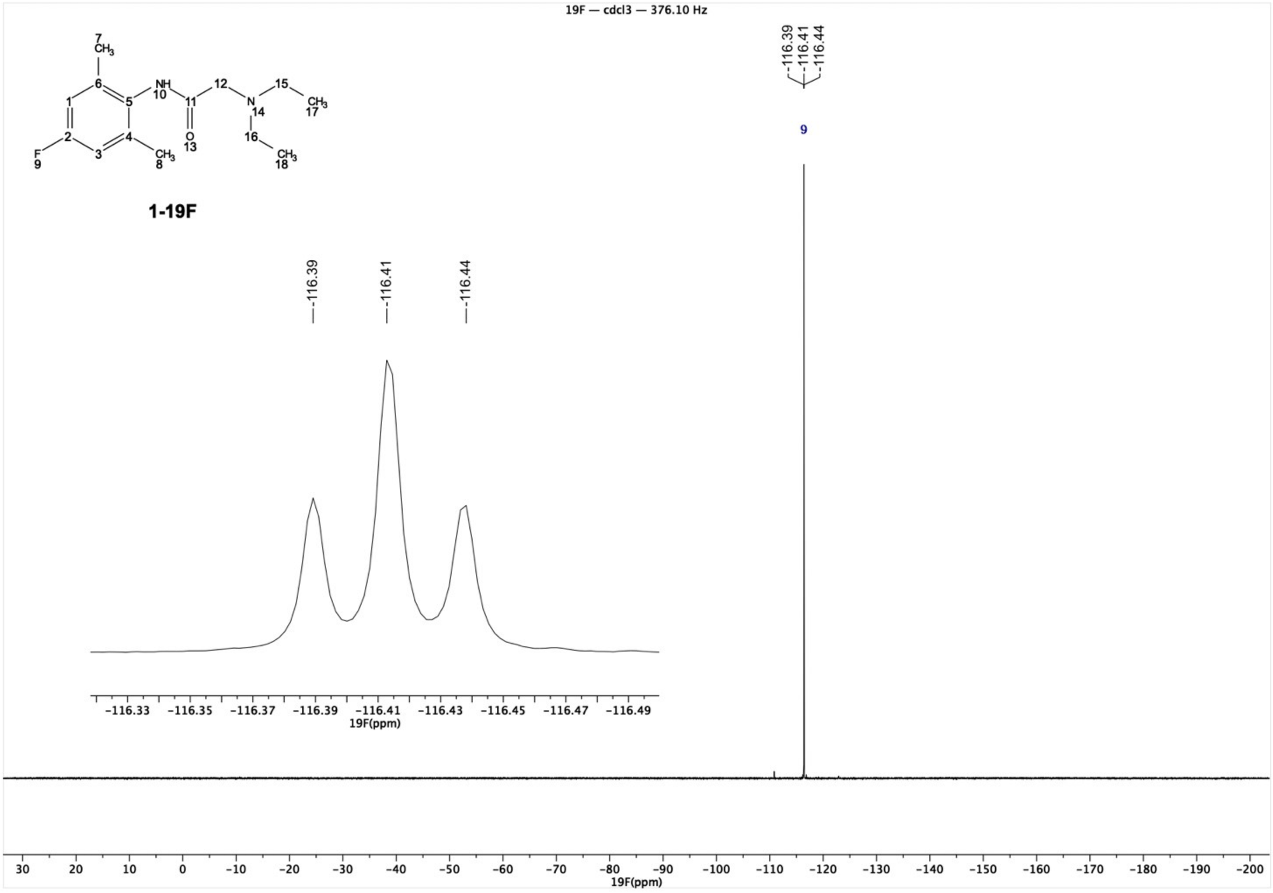

Figure 8: ^{19}F NMR Spectrum of **1-^{19}F**

2.2 Radiochemistry

2.2.1 Automated Radiosynthesis of 1-^{18}F

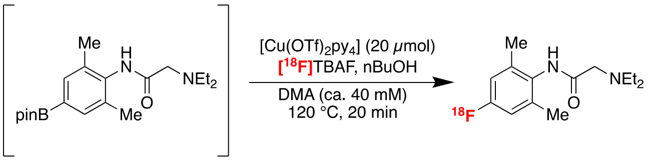

3-Bpin
(crude from Ir CHB, conditions B)
ca. 20 μmol

1-^{18}F
RCY = **0.91 ± 0.55%** (n=4)
RCP = **>99%** (n=4)
A_m = **6.23 Ci/μmol**
Synthesis time = **141 ± 19 min**

Automated radiosyntheses were conducted using a General Electric TRACERlab FX_{FN} radiosynthesis module equipped with a glass reactor vial. **3-Bpin** was generated via direct Ir-catalysed C-H borylation of **1-H** (lidocaine) under Conditions B (see Section 1.1). Radiofluoride was produced via the $^{18}O(p,n)^{18}F$ nuclear reaction using a GE PETTrace cyclotron (55 μA beam for 30 min generated ca. 1867 mCi of [^{18}F]fluoride). Radiofluoride was trapped on a QMA-light Sep-Pak cartridge preconditioned with 10 mL aqueous 0.5M KOTf. Trapped radiofluoride was eluted into the reactor with a solution of NBu_4OTf (10.4 mg, 26.5 μmol) and K_2CO_3 (0.5 mg, 3.6 μmol) in 0.5 mL H_2O. Radiofluoride was then azeotropically dried with CH_3CN via inert gas flow and heating at 100 °C. The reactor was cooled followed by sequential addition of [Cu(OTf)$_2$py$_4$] (1.0 equiv., 20 μmol, 13.6 mg) in 250 μL DMA and a solution of the crude boronate **3-Bpin** (1.0 equiv., ca. 20 μmol, 0.5M in 2-MeTHF) and nBuOH (50 μL) in DMA (200 μL) and then heated to 120 °C for 20 min. The reactor was cooled to 40 °C before reaction quenching with 3.5 mL 10mM NH_4HCO_3 in 30% CH_3CN, pH 10. Semi-preparative HPLC purification was achieved under Conditions I: Kinetex F5 5 μm, 100 Å, 250x10 mm column with pH 10 10mM NH_4HCO_3 in 30% CH_3CN buffer, isocratic method. The purified product was diluted in 50 mL H_2O and trapped on a C18 Sep-Pak C18 1 cc Vac 50 mg cartridge, which was washed with 10 mL H_2O and reformulated with 0.5 mL EtOH and 10 mL saline. The dose activity and pH were measured before confirmation of identity via analytical HPLC conditions II: Kinetex EVO 5 μM C18 100 Å 250 x 4.6 mm column with 10mM NH_4HCO_3 in 30% CH_3CN, pH 10 buffer, isocratic method. Data from four automation runs are shown in Table 2. Radiochemical yields (RCY) are reported as isolated and non-decay-corrected. Product identity was confirmed using an authentic, non-radioactive standard. Analytical NMR data, including multiplicities and coupling constants, obtained for **1-^{19}F** were consistent with this report (Figures 7, 8).[2]

Entry	Activity Yield/ mCi	RCY/%	RCP/%	Synthesis Time / min	pH
1	4.91	0.26	>99	119	5
2	28.8	1.54	>99	160	5
3	25.4	1.36	>99	126	5
4	9.00	0.48	>99	160	5

Table 2: Data Obtained from Four Automated Radiosyntheses of **1-^{18}F**.

2.2.2 HPLC Characterisation of 1-^{18}F

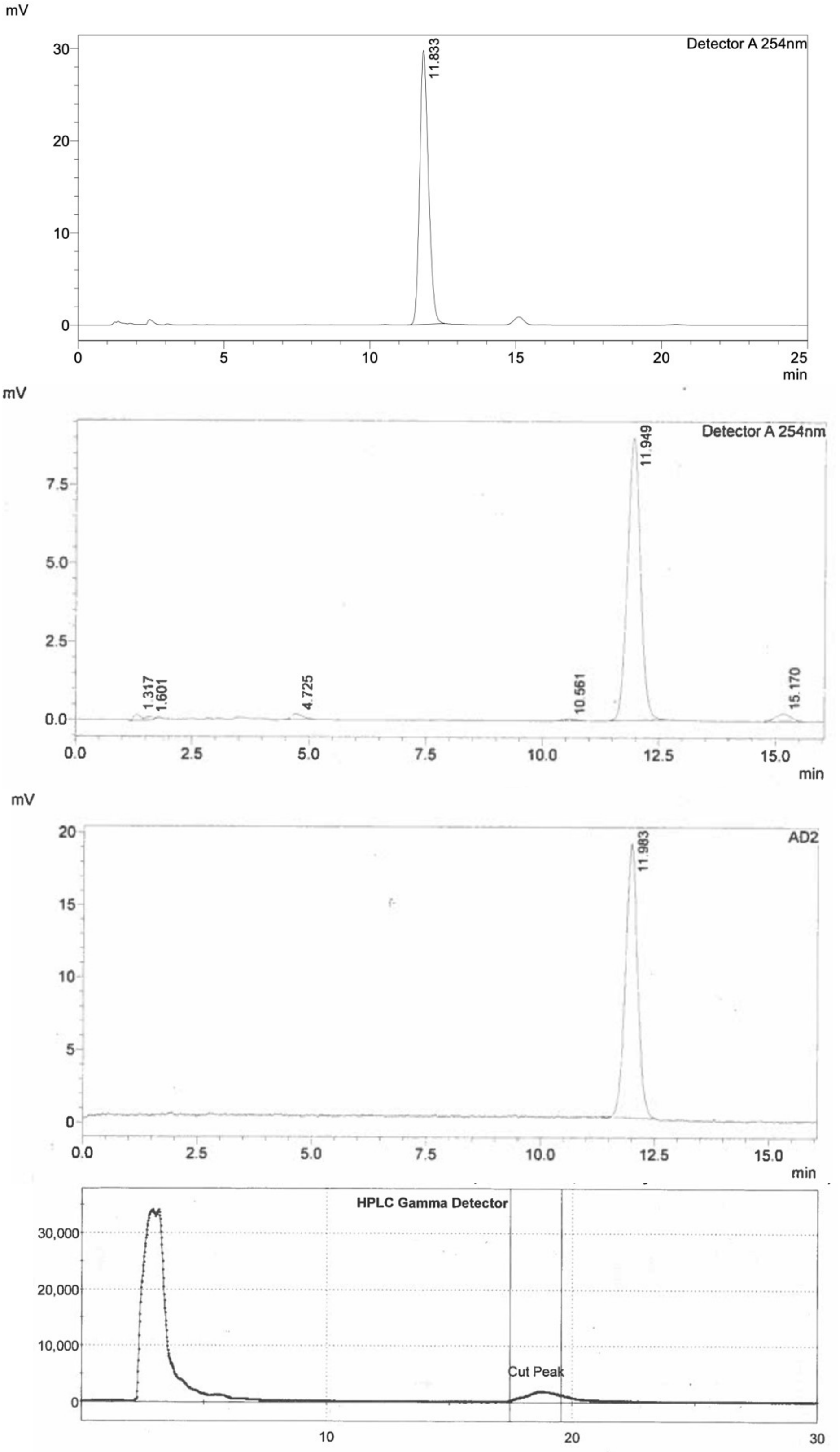

Figure 9: HPLC Chromatograms Obtained in the Characterisation of **1-^{18}F**. Upper: Analytical UV 254 nm Chromatogram of Non-Radioactive **1-^{19}F** (HPLC Conditions II). Middle Upper: Analytical UV 254 nm Chromatogram of Purified Material Collected Following Automated Radiosynthesis of **1-^{18}F** Coinjected with **1-^{19}F** (HPLC Conditions II). Middle Lower: Gamma Chromatogram of Purified Material Collected Following Automated Radiosynthesis of **1-^{18}F** Coinjected with **1-^{19}F** (HPLC Conditions II). Lower: Semi-Preparative Gamma Chromatogram Obtained During Automated Radiosynthesis of **1-^{18}F**. Cut Peak was Confirmed to be **1-^{18}F** (HPLC Conditions I).

2.2.3 Example Molar Activity Calculation of 1-^{18}F

Concentration 1-^{19}F mg/ mL	UV HPLC Peak Area (254 nm)
0.1	47683
0.01	4918
0.001	556
0.0001	84

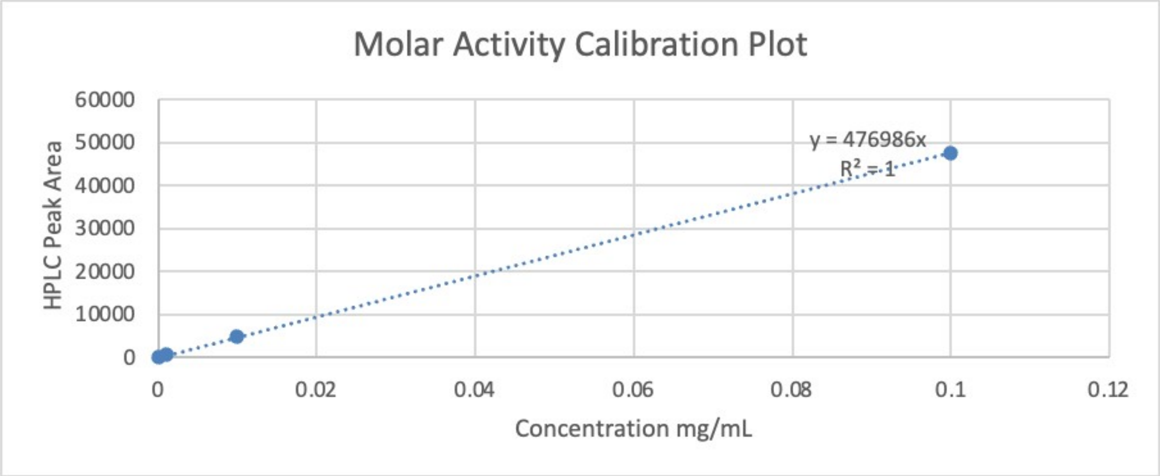

Figure 10: Selected Molar Activity Plot for Determining Decay-Corrected Molar Activity of **1-^{18}F**

3. PET Imaging Studies

3.1 General considerations:

Non-human primate and rodent PET imaging studies were conducted under the supervision of the University of Michigan and its Institutional Animal Care and Use Committee (IACUC approval number PRO00011715) according to approved protocols and all applicable federal, state, local, and institutional laws or guidelines governing animal research. Imaging studies were conducted using an MR Solutions Small Animal PET scanner.

3.2 Rodent Studies

Anaesthesia was induced in four healthy female Sprague-Dawley rats (333.75±16.3 g) using isoflurane/O_2, and anaesthesia was maintained with 2-4% isoflurane/O_2 throughout the imaging studies. A heating pad maintained body temperature. Two of the rats received an initial 2 mg mL^{-1} dose of lidocaine for a blocking study. Following anaesthetic and blocking agent administration, all four rats were injected with **1-^{18}F** (I.V. via tail vein; 0.556±0.05 µCi in 1 mL) as a bolus. All four rats were scanned for 90 min. Upon completion of the PET scans, emission data were corrected for decay, dead time, and random coincidences before reconstruction using an iterative ordered subset expectation maximisation–maximum a posteriori (MAP) method to generate reconstructed images.

3.2.1: Rodent Kinetic Data

Kinetics were obtained via region of interest analysis. Regions were drawn, and time-activity curves were generated. As shown below, calculated curves were generated and compared for various regions. This data demonstrates that for cardiothoracic regions of interest (heart wall and lungs), this agent has speedy clearance (ca. 2 min), whereas the liver, kidneys, and bladder show either much slower clearance (>90 min in the liver) or retention. With the blocking studies, the activity in the heart wall is lower (ca. 300 Bq cc^{-1}) than seen in the baseline study (ca. 480 Bq cc^{-1}).

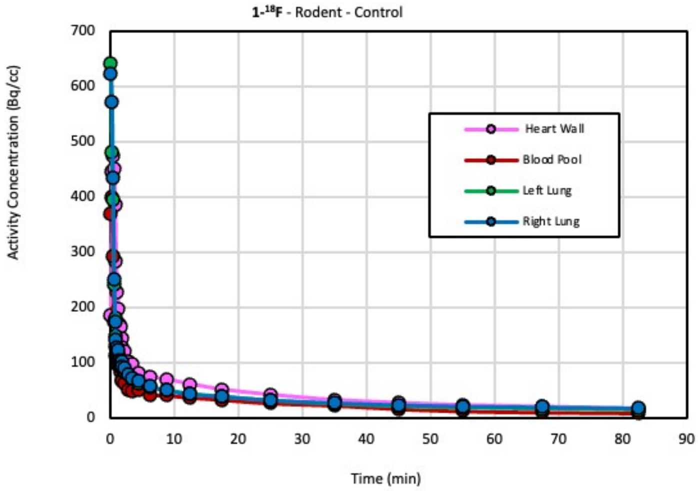
1-^{18}F - Rodent - Control
Activity Concentration (Bq/cc)
Time (min)
Heart Wall
Blood Pool
Left Lung
Right Lung

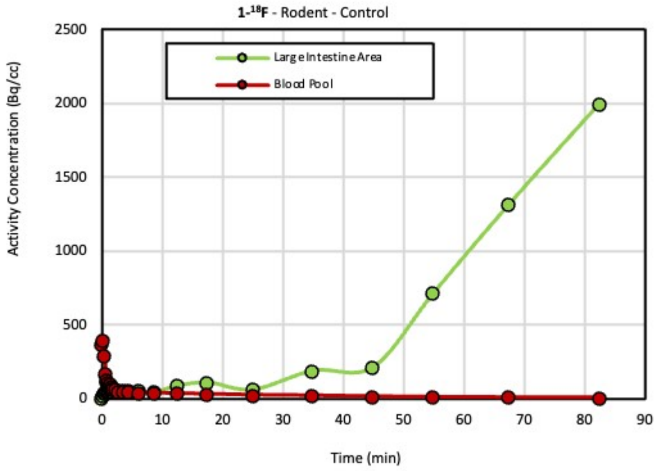
1-^{18}F - Rodent - Control
Activity Concentration (Bq/cc)
Time (min)
Large Intestine Area
Blood Pool

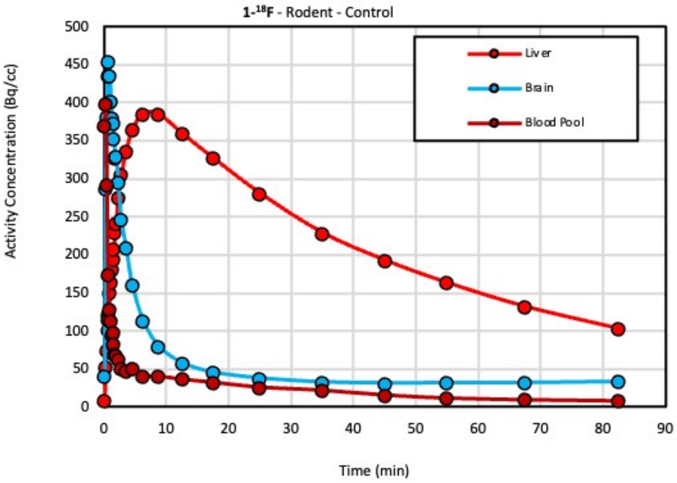
1-^{18}F - Rodent - Control
Activity Concentration (Bq/cc)
Time (min)
Liver
Brain
Blood Pool

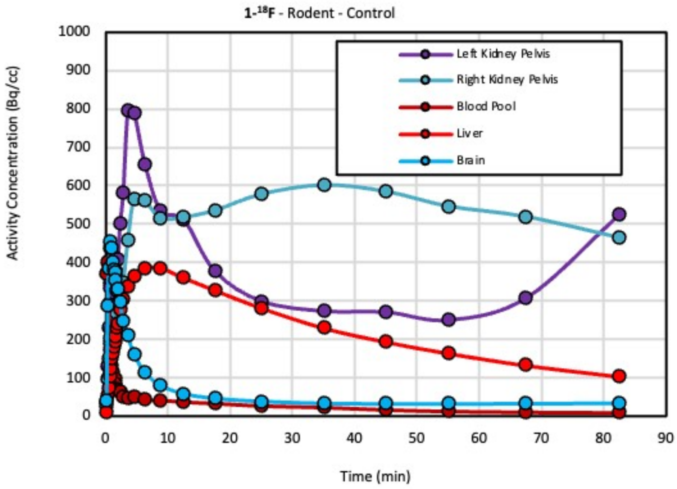
1-^{18}F - Rodent - Control
Activity Concentration (Bq/cc)
Time (min)
Left Kidney Pelvis
Right Kidney Pelvis
Blood Pool
Liver
Brain

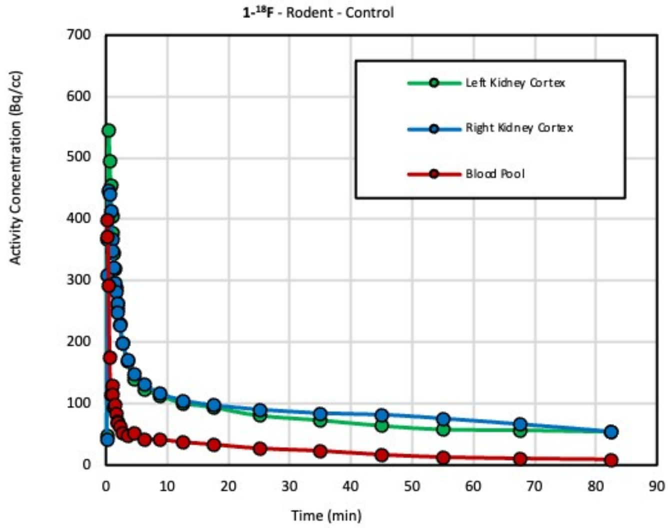
1-^{18}F - Rodent - Control
Activity Concentration (Bq/cc)
Time (min)
Left Kidney Cortex
Right Kidney Cortex
Blood Pool

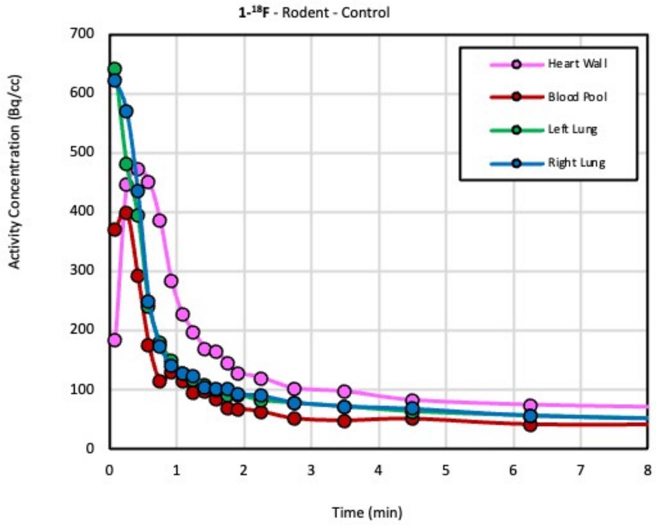
1-^{18}F - Rodent - Control
Activity Concentration (Bq/cc)
Time (min)
Heart Wall
Blood Pool
Left Lung
Right Lung

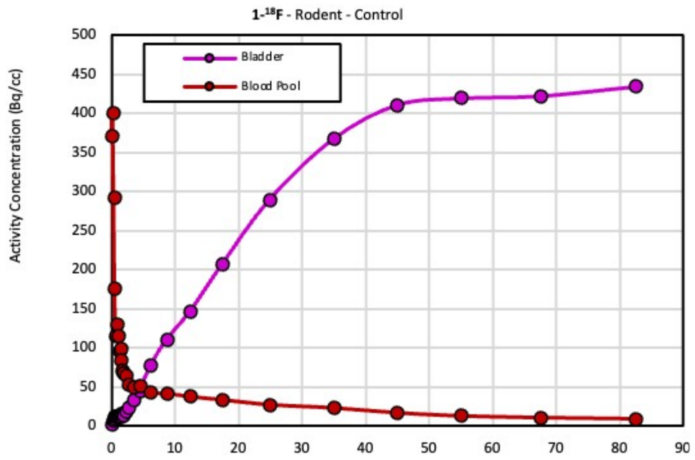
1-18F - Rodent - Control
Activity Concentration (Bq/cc)
Bladder
Blood Pool

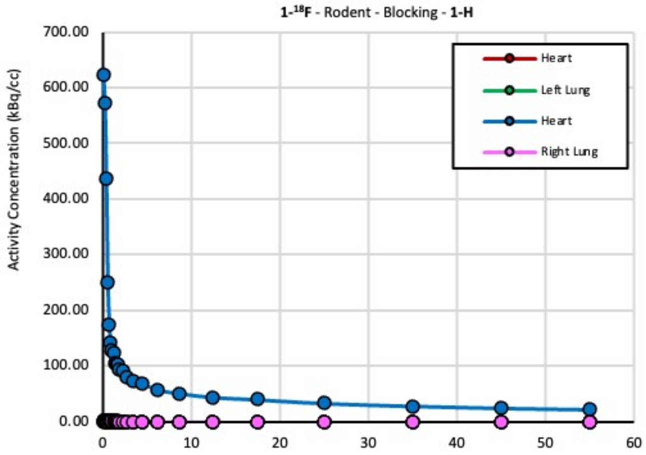
1-18F - Rodent - Blocking - 1-H
Activity Concentration (kBq/cc)
Heart
Left Lung
Heart
Right Lung

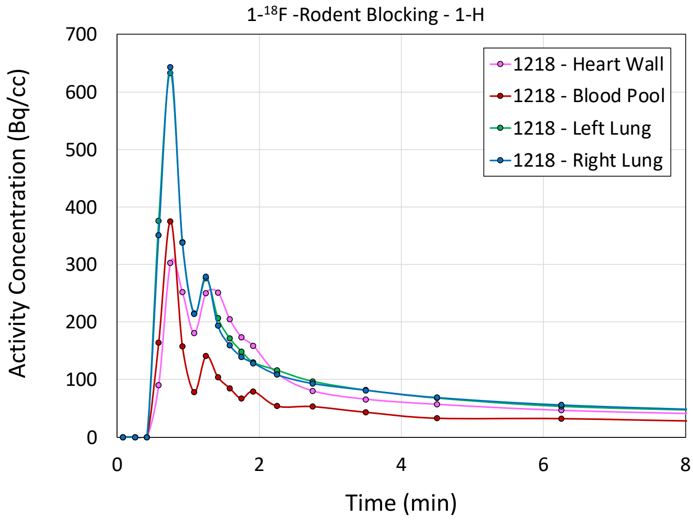
1-18F -Rodent Blocking - 1-H
Activity Concentration (Bq/cc)
1218 - Heart Wall
1218 - Blood Pool
1218 - Left Lung
1218 - Right Lung
Time (min)

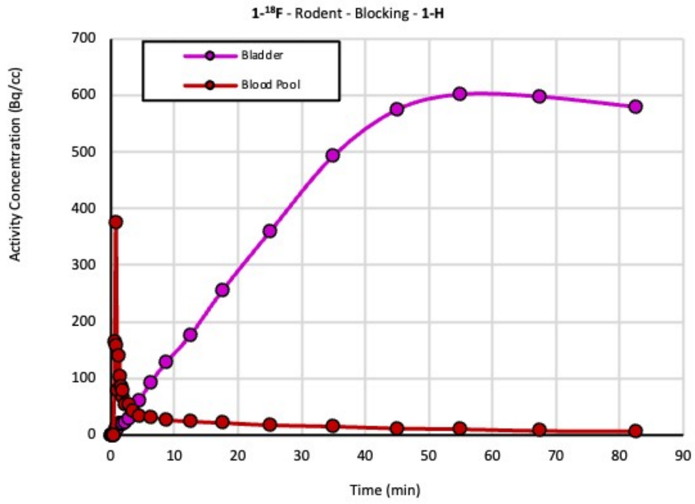
1-18F - Rodent - Blocking - 1-H
Activity Concentration (Bq/cc)
Bladder
Blood Pool
Time (min)

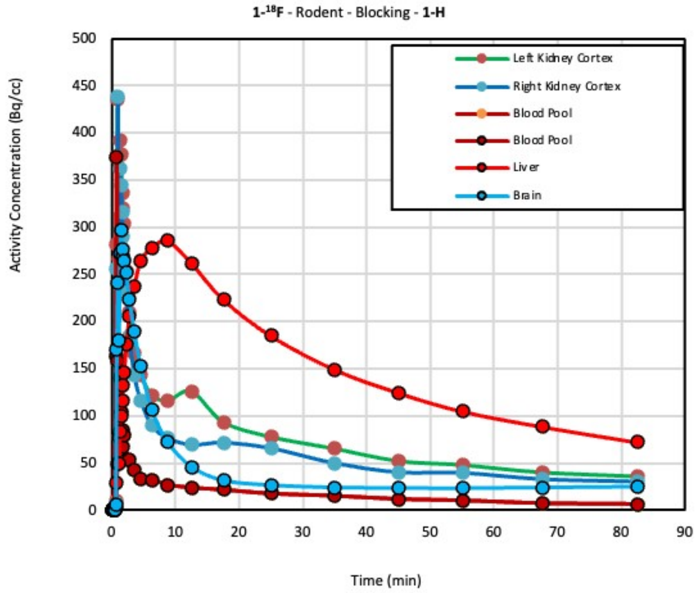
1-18F - Rodent - Blocking - 1-H
Activity Concentration (Bq/cc)
Left Kidney Cortex
Right Kidney Cortex
Blood Pool
Blood Pool
Liver
Brain
Time (min)

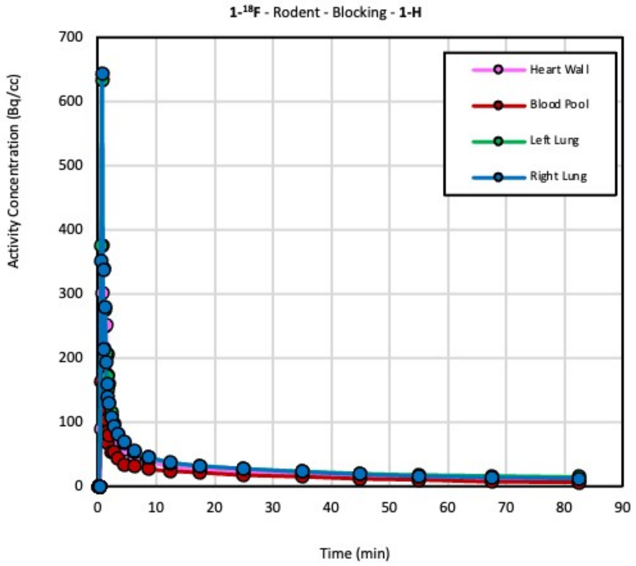
1-18F - Rodent - Blocking - 1-H
Activity Concentration (Bq/cc)
Heart Wall
Blood Pool
Left Lung
Right Lung
Time (min)

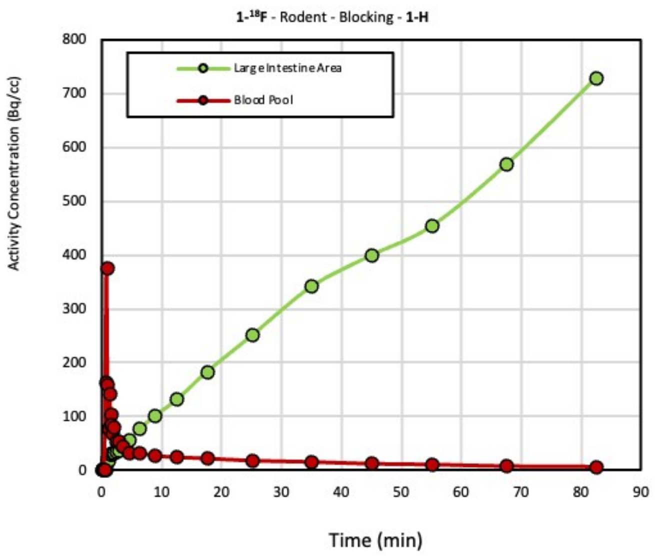

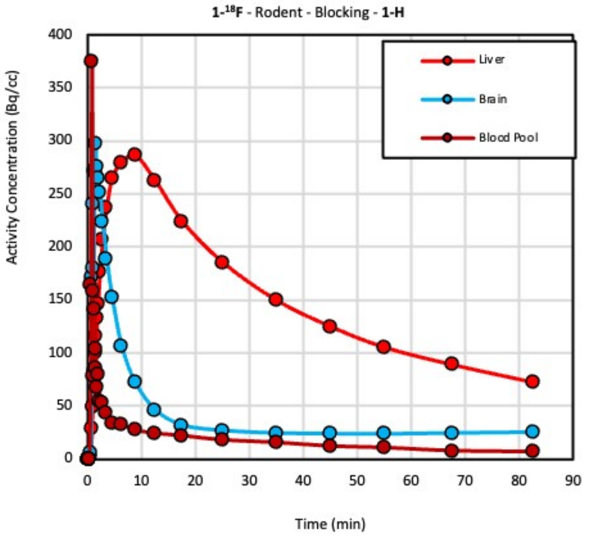

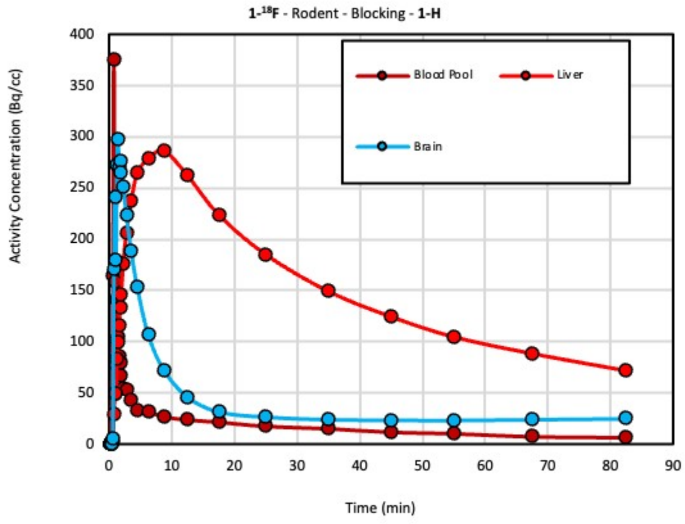

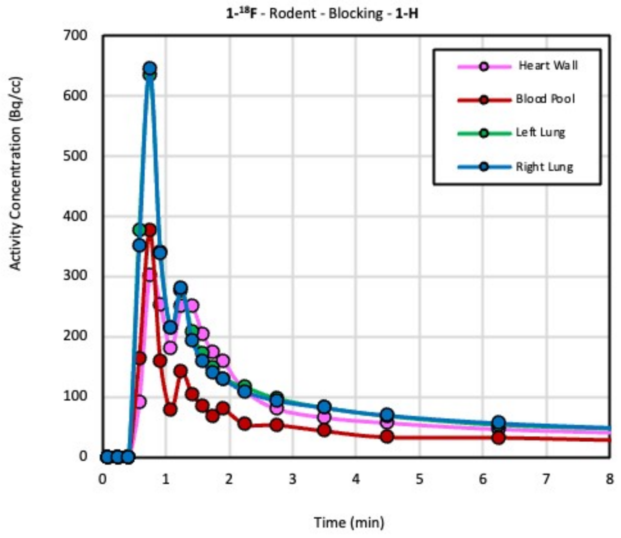

3.3 Non-Human Primate Studies

Non-human primate (NHP) studies were conducted in duplicate with a female rhesus monkey weighing 6.541 lb. An intramuscular injection of 0.15 mL of a 100 mg/mL Telazol® solution was administered for sedation. Isoflurane was used for sustained anaesthesia. A 4.0 mm endotracheal tube was inserted, and the primate was placed on a ventilator. A 24 g I.V. catheter was placed in the left saphenous vein, and 4.07 mCi of **1-^{18}F** was administered in 8.0 mL of 5% EtOH in saline. PET Imaging was conducted over 90 min. Images obtained from this study are shown below. As the rodent data showed, uptake in the heart wall was observed at around 2-4 min post-injection. However, this activity is not observed in the summed images shown in Figure 11, and uptake was insignificant during the 90 min scan. Due to the lack of heart retention, a blocking study was not performed.

3.3.1 Non-Human Primate Images

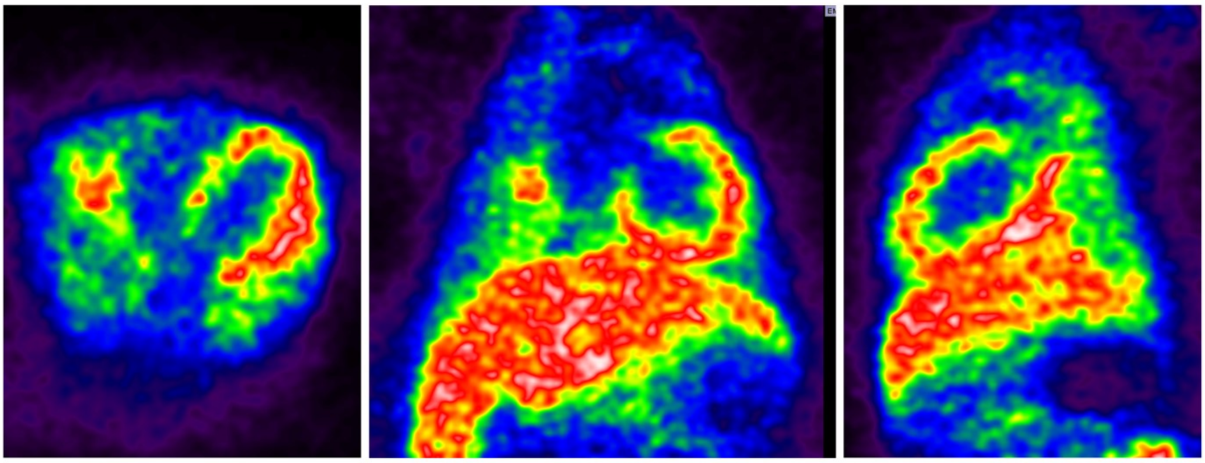

Figure 11: NHP Summed Image Frames 4-7 Corresponding to 105-270 s with Rainbow Scale Table Maximum Intensity Projection (Baseline Control Study).

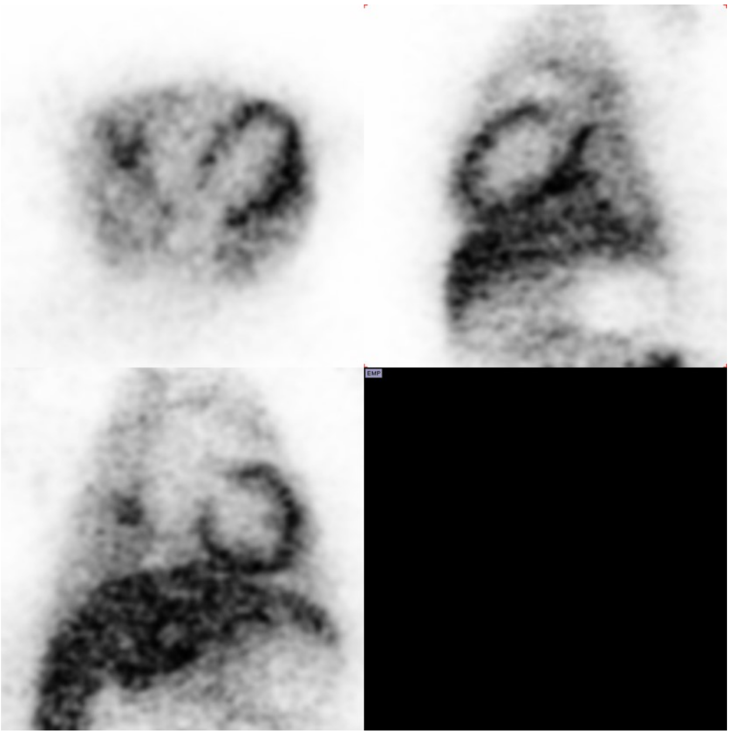

Figure 12: NHP Summed Image Frames 4-7 Corresponding to 105-270 s in Grayscale with Table Maximum Intensity Projection (Baseline Control Study).

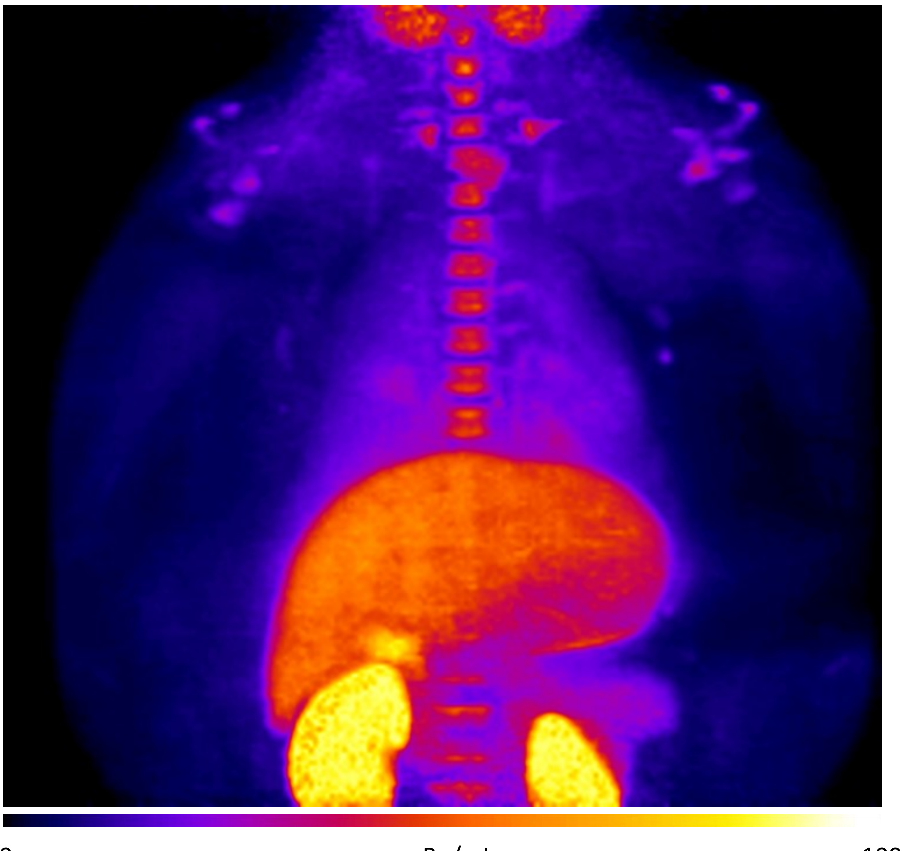

Figure 13: Non-Human Primate Total Summed Image Frames with Fire Table Maximum Intensity Projection (Baseline Control Study).

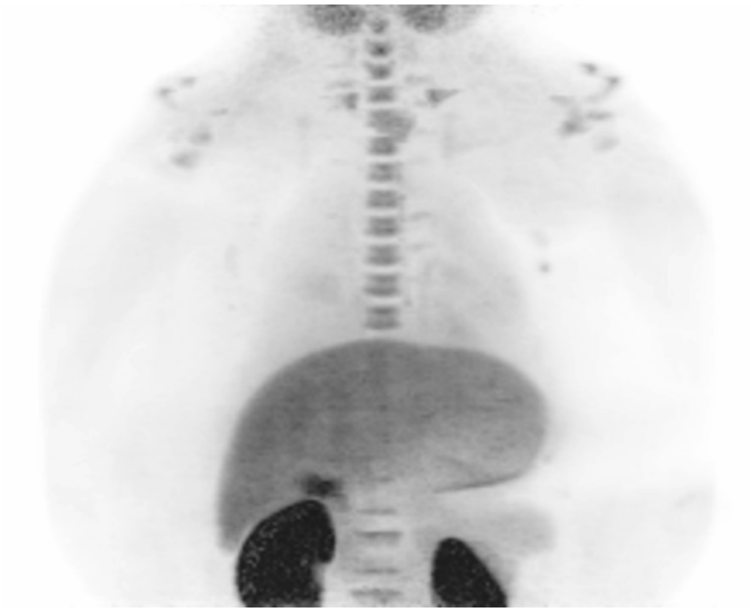

Figure 14: Non-Human Primate Total Summed Image Frames in Grayscale with Maximum Intensity Projection

4. References

1 Tajuddin H., Harrisson P., Bitterlich B., Collings J. C., Sim N., Batsanov A. S., Cheung M. S., Kawamorita S., Maxwell A. C., Shukla L., Morris J., Lin Z., Marder T. B., and Steel P. G., *Chem. Sci.,* 2012, **39**, 3505–3515.
2. Wright J. S., Sharninghausen L. S., Preshlock S., Brooks A. F., Sanford M. S., Scott P. J. H., *J. Am. Chem. Soc.*, 2021, **143**, 6915-6921.
